# Early nutrition is safe and does not increase complications after upper gastrointestinal bleeding—a systematic review and meta-analysis of randomized controlled trials

**DOI:** 10.1038/s41598-024-61543-z

**Published:** 2024-05-10

**Authors:** Mahmoud Obeidat, Brigitta Teutsch, Diana-Elena Floria, Dániel Sándor Veres, Péter Hegyi, Bálint Erőss

**Affiliations:** 1 Centre for Translational Medicine, Semmelweis University, Budapest, 1085 Hungary; 2 Institute for Translational Medicine, Medical School, University of Pécs, Pécs, 7623 Hungary; 3 Grigore T. Popa University of Medicine and Pharmacy, 700115 Iași, Romania; 4 Department of Biophysics and Radiation Biology, Semmelweis University, Budapest, 1085 Hungary; 5 Institute of Pancreatic Diseases, Semmelweis University, Budapest, 1083 Hungary

**Keywords:** Hepatology, Oesophagogastroscopy, Gastrointestinal bleeding, Nutrition disorders, Ulcers

## Abstract

Despite a lack of evidence, patients are often not fed for 48–96 h after upper gastrointestinal bleeding (UGIB); however, many trials have demonstrated the benefits of early nutrition (EN). We conducted a meta-analysis of randomized controlled trials (RTCs) to evaluate the outcomes of EN compared to delayed nutrition (DN) after UGIB. The protocol was registered on PROSPERO (CRD42022372306). PubMed, Embase, CENTRAL, Scopus, and Web of Science were searched on the 27th of April 2024 to identify eligible RCTs. The primary outcomes were early (within 7 days) and late (within 30–42 days) mortality and rebleeding. Pooled risk ratios (RR), mean differences (MD), and corresponding 95% confidence intervals (CI) were calculated using a random-effects model. A total of 10 trials with 1051 patients were included in the analysis. Early mortality was not significantly different between the two groups (RR 1.20, CI 0.85–1.71, *I*^2^ = 0%), whereas late mortality was reduced to a clinically relevant extent in the EN group (RR 0.61, CI 0.35–1.06, *I*^2^ = 0%). When comparing the two groups, we found no significant difference in terms of early and late rebleeding (RR 1.04, CI 0.66–1.63, *I*^2^ = 0% and RR 1.16, CI 0.63–2.13, *I*^2^ = 0%, respectively). Our analysis also showed that the length of hospital stay was reduced in the EN group compared to the DN group (MD −1.22 days, CI: −2.43 to −0.01, *I*^2^ = 94%). In conclusion, compared with DN, EN (within 24 h) appears to be a safe intervention and could reduce the length of hospital stay without increasing the risk of complications after UGIB.

## Introduction

Upper gastrointestinal bleeding (UGIB) is a common medical emergency with severe consequences if not managed appropriately. It can be triggered by a variety of underlying causes, such as peptic ulcers and varices. Its incidence is increasing unacceptably, with mortality rates ranging from 2 to 10%^1–4^. Regardless of the source, bleeding can lead to malnutrition and other complications that can prolong hospitalization and increase mortality. Therefore, providing adequate nutrition to patients with UGIB is crucial.

Early nutrition (EN) has been suggested to improve outcomes by reducing the risk of infections^5^ and maintaining gut mucosal integrity. On the other hand, delayed nutrition (DN) has been considered to minimize the risk of rebleeding and other complications arising from introducing food or nutrients too soon after an episode of bleeding^6,7^. However, it can also lead to malnutrition and delayed recovery.

Based on current research findings, patients at high risk of rebleeding are recommended to abstain from eating and stay hospitalized for at least 48–72 h following endoscopic treatment. During this period, many high-risk lesions are expected to become low-risk, and the majority of rebleeding incidents tend to occur^8,9^. However, The optimal time to start feeding remains a controversial topic, and the nutrition strategy should be based on endoscopic findings in patients with UGIB^6^. Patients who have low-risk endoscopic findings, such as clean-based ulcers, can typically resume a regular diet shortly after the endoscopic procedure and may be discharged if there are no other reasons for hospitalization^10^. However, those with higher-risk endoscopic findings, even after undergoing endoscopic therapy, may require feeding within 72 h, although the exact timing and specific dietary recommendations are uncertain^9,11,12^. Individual patient factors, such as severity of bleeding, comorbidities, and risk of complications, should also be considered when making treatment decisions. In this context, it is essential to weigh up the potential benefits and risks of early versus delayed nutrition.

Some studies showed that EN could be beneficial in reducing complications in patients with UGIB^13–15^; however, others favored DN^7,16^. A previous meta-analysis by Zhang et al.^17^ set out to investigate this clinical question but included only five trials. In contrast, we evaluated a broader spectrum of outcomes and analyzed the early and late rebleeding and mortality separately. In addition, we included five more trials compared to the previously investigated ones. Therefore, we meta-analyzed randomized controlled trials (RCTs) to assess the efficacy and safety of EN compared to DN and grouped them by source of bleeding.

## Methods

Our meta-analysis was conducted following the recommendations of the Cochrane Handbook and the Preferred Reporting Items for Systematic Reviews and Meta-Analyses (PRISMA) 2020 guidelines (Supplementary Material Table S1)^18,19^. The study protocol was registered on PROSPERO (CRD42022372306) in advance, and we fully adhered to it^20^.

### Eligibility criteria

Only RCTs were included in our analysis. We applied the PICO (patients, intervention, comparison, outcome) framework to establish our eligibility criteria^21^; patients were upper gastrointestinal bleeders, including variceal and non-variceal bleeding sources, and the intervention was early nutrition (EN) compared to delayed nutrition (DN). Our primary outcomes were early (within 7 days) and late (within 30–42 days) mortality and rebleeding, whereas the length of hospital stay (LOS), transfusion requirement, transfusion rate, and ICU admission were our secondary endpoints. As for post-hoc analysis, we included bacterial infection, new-onset ascites, and hepatic encephalopathy as additional outcomes. Data on rebleeding and mortality outcomes were combined if reported within three, five, or seven days (early: within 7 days), and likewise for late outcomes, which were pooled together if reported within 30 or 42 days. Any definition for early and delayed nutrition was accepted as specified by the included studies.

### Information sources

Our systematic search was conducted in five main databases: Embase, MEDLINE (via PubMed), Cochrane Central Register of Controlled Trials (CENTRAL), Scopus, and Web of Science, from inception to 10th November 2022, and we updated the search on the 27th of April 2024. No language or other restrictions were applied. In addition, a backward and forward citation search was performed using a reference-checking tool to identify all potential references that met our eligibility criteria.

### Search strategy

Our search key included three domains. The first domain focused on early and delayed nutrition, whereas the second domain included sources of gastrointestinal bleeding (GIB) and their synonyms. The third domain focused on the concept of randomization, as we included only RCTs. For a detailed search key, see Supplementary Material Table S2.

### Screening and selection

After a systematic search, the resulting articles were imported into a reference management program (EndNote 20.1, Clarivate Analytics). Duplicate articles with overlapping publication years, authors, and titles were eliminated automatically and manually. Screening and selection were performed by two independent reviewers (M.O. and D.E.F.), first by title and abstract, and then by full text (considering the eligibility criteria). Cohen's kappa coefficient was calculated at both levels of selection to measure the inter-reviewer reliability^22^. In case of any disagreement, consensus was reached after discussion with the corresponding author (B.E.).

### Data extraction

Relevant data from the eligible studies were extracted independently by two authors (M.O. and D.E.F.). Disagreements were resolved by involving the corresponding author (B.E.). All data were manually collected and entered into an Excel spreadsheet (Office 365, Microsoft, Redmond, WA, USA) in preparation for analysis.

### Data items

The following data were extracted: first author, year of publications, digital object identifier, study population, geographic location, study design and period, basic demographics (sex and age), source of bleeding, and bleeding severity scores, including Forrest classification for peptic ulcer bleeding (PUB). Child–Pugh score, Model for end-stage liver disease (MELD) score, and size of esophageal varices were also extracted. In addition, we extracted data on the total number of patients in each arm (early and delayed nutrition), the definition of the outcomes of interest, the definition of the interventions in terms of timing and type of diet used.

### Risk of bias assessment and quality of evidence

Two independent authors (M.O. and D.E.F.) assessed the methodological quality of each trial using the revised tool for assessing the risk of bias (ROB 2) recommended by the Cochrane Handbook^23^. A third reviewer resolved potential disagreements (B.T.). Accordingly, the following potential sources of bias were evaluated: bias due to the randomization process, bias due to deviations from the planned interventions, bias due to missing outcome data, and bias in the measurement of the outcome or the selection of the reported results. We used the robvis (Risk-Of-Bias VISualization) tool to create risk-of-bias plots^24^.

To assess the quality of evidence for our results, we followed the Grading of Recommendations Assessment, Development, and Evaluation (GRADE) approach^25^, and used the GRADEpro Guideline Development Tool (software) to produce the summary tables of findings. The determinants were study design, risk of bias, inconsistency, indirectness, and imprecision.

### Statistical synthesis

The minimum number of studies needed to perform the meta-analysis was three. As we assumed considerable between-study heterogeneity in all cases, a random-effects model was used to pool effect sizes. Risk ratios (RR) with 95% confidence intervals (CI) were used to measure effect size for dichotomous outcomes. For continuous outcomes, the mean difference (MD) was used to measure effect size. To calculate study RR and pooled RR, the total number of patients and the total number of patients with the event of interest in each group were extracted separately from the studies. We reported the results as the risk of an event of interest in the EN group versus the risk of an event of interest in the DN group. To calculate the pooled MD, the mean and standard deviation (SD) were extracted from each study. The studies by Jatin et al.^15^ and Gong et al.^16^ reported the LOS using median and SD; therefore, we performed the analysis without them, and included them in a separate analysis assuming a symmetrical distribution where the mean was equal to the median (as the other study reported using mean and SD, we assumed that this was acceptable). The MD was expressed as the mean of the EN group minus the mean of the DN group.

The pooled RR was calculated using the Mantel–Haenszel method^26,27^. The exact Mantel–Haenszel method (without continuity correction) was used to handle zero cell counts as recommended^28^. The inverse variance weighting method was used to calculate the pooled MD. We used a Hartung–Knapp adjustment for CIs^29,30^. To estimate the heterogeneity variance measure (${\tau }^{2}$) for RR calculation, the Paule–Mandel method^31^ was used. For MD calculation, the restricted maximum-likelihood estimator was used with the Q profile method for CI. Prediction interval calculations were based on t-distribution.

Results were considered statistically significant if the CI did not contain the null value. We summarized the findings for the meta-analysis in forest plots. In forest plots, for a cell count of zero, the RR of each study with 95% CI was calculated by adding 0.5 as continuity correction (it was used only for the forest plots). Where applicable (the study number was large enough and not too heterogeneous), we reported the prediction intervals for the results. In addition to ${\tau }^{2}$, heterogeneity was assessed using Higgins and Thompson *I*^2^ statistics^32^. The statistical analysis of the data was conducted using the R software (R Core Team, 2019, Vienna, Austria).

For subgroup analyses, we used a fixed-effects “plural” model (mixed-effects model). We assumed different ${\tau }^{2}$ values in the subgroups. To assess the difference between the subgroups, the Cochran’s Q test was used. The null hypothesis was rejected at a 5% significance level. The subgroup analysis by source of bleeding was planned before the data collection. Publication bias in small studies was assessed by visual inspection of Funnel-plots and calculation of p-values for Harbord (for RR)^33^ and Egger (for MD) tests^34^. We planned to assume potential bias in small studies if the *p*-value was less than 10%. (Although we kept in mind that the test had limited diagnostic assessment below 10 studies). Potential outlier publications were explored using different influence measures and plots following the recommendation of Harrer et al.^35^.

All statistical analyses were performed with *R* (v4.1.2) using the *meta*^36^ (v6.1.0) package for basic meta-analysis calculations and plots, and the *dmetar*^37^ (v0.0.9000) package for additional influential analysis calculations and plots.

### Ethical approval

No ethical approval was required for this systematic review with meta-analysis, as all data were already published in peer-reviewed journals. No patients were involved in our study design, conduct, or interpretation. The datasets used in this study can be found in the full-text articles included in the systematic review and meta-analysis.

## Results

### Search and selection

**Figure 1:**
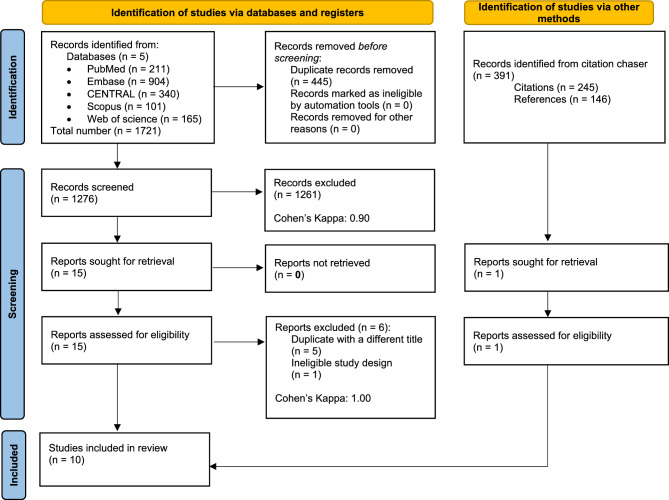
PRISMA flowchart of the article selection process.

Altogether, 1721 records were identified in the five databases, 904 in Embase, 211 in MEDLINE (via PubMed), 340 in CENTRAL**,** 101 in Scopus, and 165 in Web on Science. After duplicate removal, 1276 records remained for title and abstract selection. A total of 15 studies were assessed for full-text eligibility, of which six were excluded^38–43^. Five of the six excluded studies were duplicates but with different titles^38,40–43^, and one had an ineligible study design^39^. In addition, we identified 391 records through citation chasing, but only one study was sought for retrieval and was eligible for data extraction^44^. For more details on our search and selection process, see the PRISMA flowchart (Fig. 1).

### Basic characteristics of studies included

Our study consisted of 10 RCTs with a total of 1051 patients^5,7,13–16,44–47^. One of these studies was published as a conference abstract^47^. Five of the 10 studies focused on patients with non-variceal upper gastrointestinal bleeding (NVUGIB), mainly PUB^13,16,45–47^, whereas the other five focused on patients with VUGIB^5,7,14,15,44^. The studies were conducted in various geographical locations, five in Asia, three in Europe, one in Africa, and one in North America. More information on the trials, including their basic characteristics, can be found in (Table 1).

**Table 1:** Basic characteristics of included studies.

Study (year)	Study site	Bleeding source	Hemostatic Intervention	Number of patients (female %)	Age (years), mean ± SD	Child–Pugh Score (A/B/C) (mean score ± SD)	Size of the Esophageal Varices (F1/F2/F3) (MELD Score ± SD)	Time of feeding	
								EN	DN
A. Studies with variceal upper gastrointestinal bleeding									
Sidhu et al. 2019^5^	India	AVB	EVBL	EN: 52 (11.5)	EN: 51.48 ± 11.22	EN: 12/36/4 (9.1 ± 1.2)	EN: 0/40/12 (14.6 ± 8.2)	Liquid diet after 1 h, regular diet after 4 h	Liquid diet after 4 h, soft diet after 48 h, regular diet after 72 h
				DN: 49 (22.4)	DN: 47.21 ± 12.6	DN: 9/34/6 (8.6 ± 1.5)	DN: 0/38/11 (13.8 ± 6.6)		
Goda et al. 2018^44^	Egypt	AVB	EVBL or injection sclerotherapy	EN: 45 (20)	EN: 57.56 ± 8.7	EN: 9/25/11	EN: 4/14/27	4 h after endoscopic intervention	48 h after endoscopic management
				DN: 45 (28.8)	DN: 56.96 ± 8.8	DN: 10/24/11	DN: 3/13/29		
Gin-Ho Lo et al. 2015^14^	Taiwan	GEVB	EVBL, histoacryl injection for GVB	EN: 36 (13.8)	EN: 47.5 ± 12.6	EN: 10/14/12 (7.6 ± 1.8)	EN: 6/22/8 (12.4 ± 3.7)	4 h after endoscopic intervention	48 h after endoscopic intervention
				DN: 34 (17.6)	DN: 53.2 ± 11.8	DN: 11/17/6 (8.2 ± 2.2)	DN: 5/22/7 (13.3 ± 4.2)		
Ledinghen et al. 1997^7^	France	AVB	Emergency sclerotherapy or VBL	EN: 12 (33.3)	EN: 59 ± 11.8	EN: 1/5/6 (10.1 ± 2.7)	EN: 1/9/2	Within 24 h	After 72 h
				DN: 10 (10)	DN: 52.3 ± 10	DN: 1/4/5 (10.4 ± 2.7)	DN: 0/8/2		
Jatin et al. 2022^15^	India	AVB	EVBL, glue injection	EN: 40	EN: 39.4 ± 12.3	EN: 0/33/7 (8,7 ± 1.3)	EN: NA (13 ± 3.4)	Liquid diet after 1 h for 6 h with soft diet	Only liquid diet for 48 h then solid diet started
				DN: 40	DN: 42.6 ± 10.5	DN: 0/28/10 (8.8 ± 1.6)	DN: NA (14.1 ± 4.5)		

Study (year)	Study site	Bleeding source	Hemostatic intervention	Number of patients (female %)	Age (years), mean ± SD	Forrest classification Ia/Ib/IIa/IIb/III	Time of feeding		
							EN	DN	
B. Studies with non-variceal upper gastrointestinal bleeding									
Gong et al. 2020^16^	Korea	PUB with HRS	Various methods^a^	EN: 103 (14.6)	EN: 61.2 ± 17	EN: 14/36/50/3/0	24 h after successful hemostasis	48 h after successful hemostasis	
				DN: 106 (21.7)	DN: 61.6 ± 15.9	DN: 17/36/49/4/0			
Khoshbaten et al. 2013^13^	Iran	PUB	Endoscopic sclerotherapy or APC or both	EN: 50 (38)	EN: 56.6 ± 17.8	EN: 0/0/43/7/0	6–12 h after endoscopic treatment	72 h after endoscopic treatment	
				DN: 50 (36)	DN: 58.7 ± 18.1	DN: 0/0/46/4/0			
Laine et al. 1992^45^	US	PUB, MWT	No endoscopic intervention	EN: 130	NA	NA	Immediate	After 36 h	
				DN: 128					
Ledinghen et al. 1998^46^	France	PUB	Emergency endoscopic injection with adrenaline (sclerotherapy)	EN: 12	EN: 75 (33–91)^b^	EN: 0/4/2/6/0	Within 24 h	After 72 h	
				DN: 14	DN; 69 (46–92)^b^	DN: 0/7/3/4/0			
Hepworth et al. 1995^c^^47^	UK	PUB	Adrenaline and ethanolamine sclerotherapy	EN: 47	NA	NA	Normal diet and milk after hemostasis	After 24 h	
				DN: 48					

*AVB* acute variceal bleeding, *GEVB* gastroesophageal variceal bleeding, *PUB* peptic ulcer bleeding, *HRS* high-risk stigmata, *MWT* Mallory-Weiss tears, *EVBL* esophageal variceal band ligation, *GVB* gastric variceal bleeding, *APC* argon plasma coagulation, *EN* early nutrition, *DN* delayed nutrition, *SD* standard deviation.

^a^Gong et al. 2020 used various hemostatic methods, including thermal, mechanical, injection, and combination therapy.

^b^Ledinghen et al. 1998 reported the median age and range.

^c^Conference abstract.

### Early rebleeding (within 7 days)

**Figure 2:**
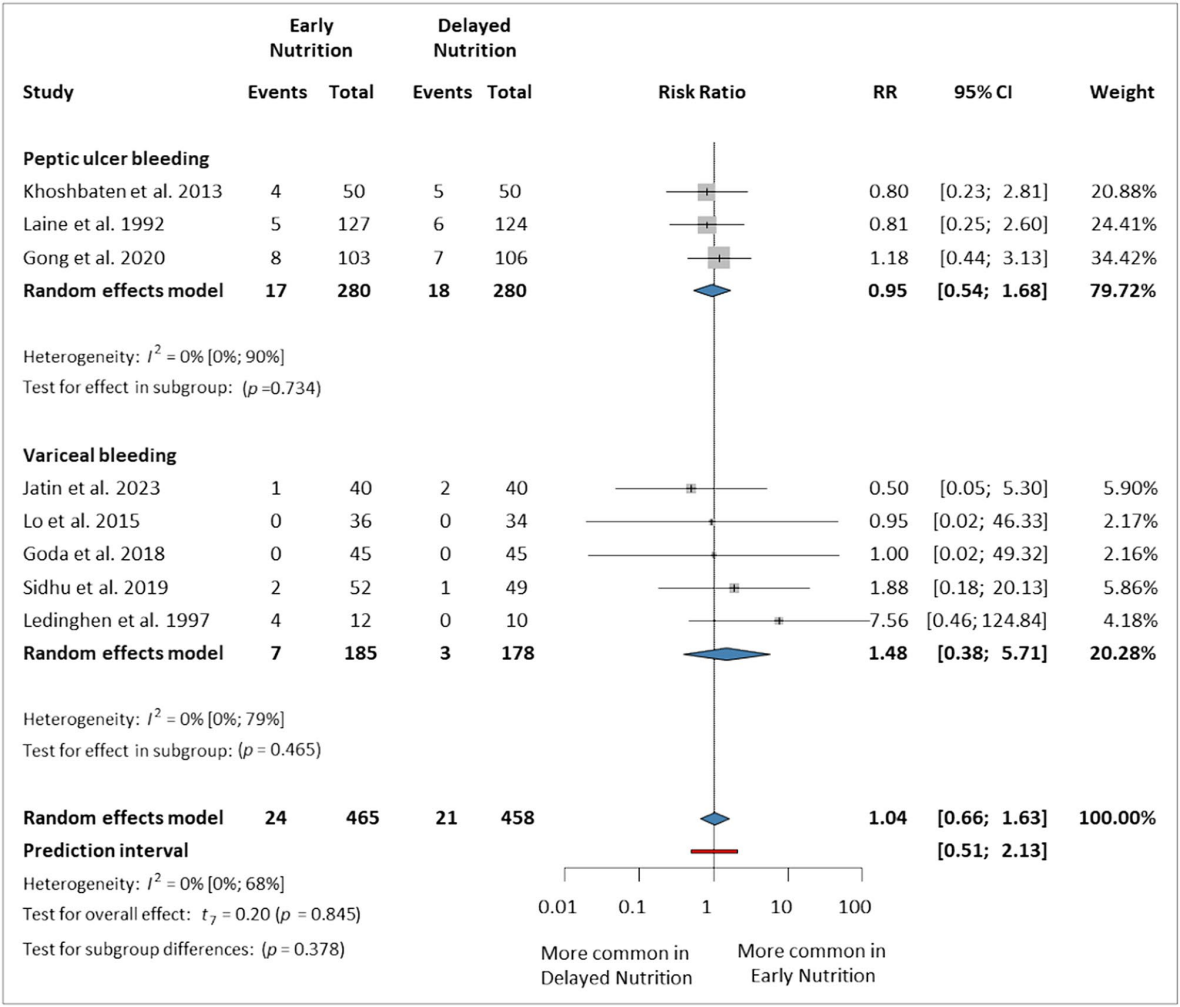
Forest plot demonstrating the effect of early versus delayed nutrition on early rebleeding (within 7 days) after upper gastrointestinal bleeding. *RR* risk ratio, *CI* confidence interval.

We analyzed eight trials^5,7,13–16,44,45^ that reported rebleeding within seven days, involving 923 patients (465 in the EN group and 458 in the DN group). In the VUGIB subgroup, our analysis showed no significant difference between the two groups (RR 1.48, 95% CI 0.38–5.71), similarly in the PUB subgroup (RR 0.95, 95% CI 0.54–1.68). Overall, EN did not significantly or relevantly increase the risk of early rebleeding compared to DN (RR 1.04, 95% CI 0.66–1.63, *p* = 0.845, *I*^2^ = 0%, 95% CI 0–68%) (Fig. 2).

### Late rebleeding (within 30–42 days)

**Figure 3:**
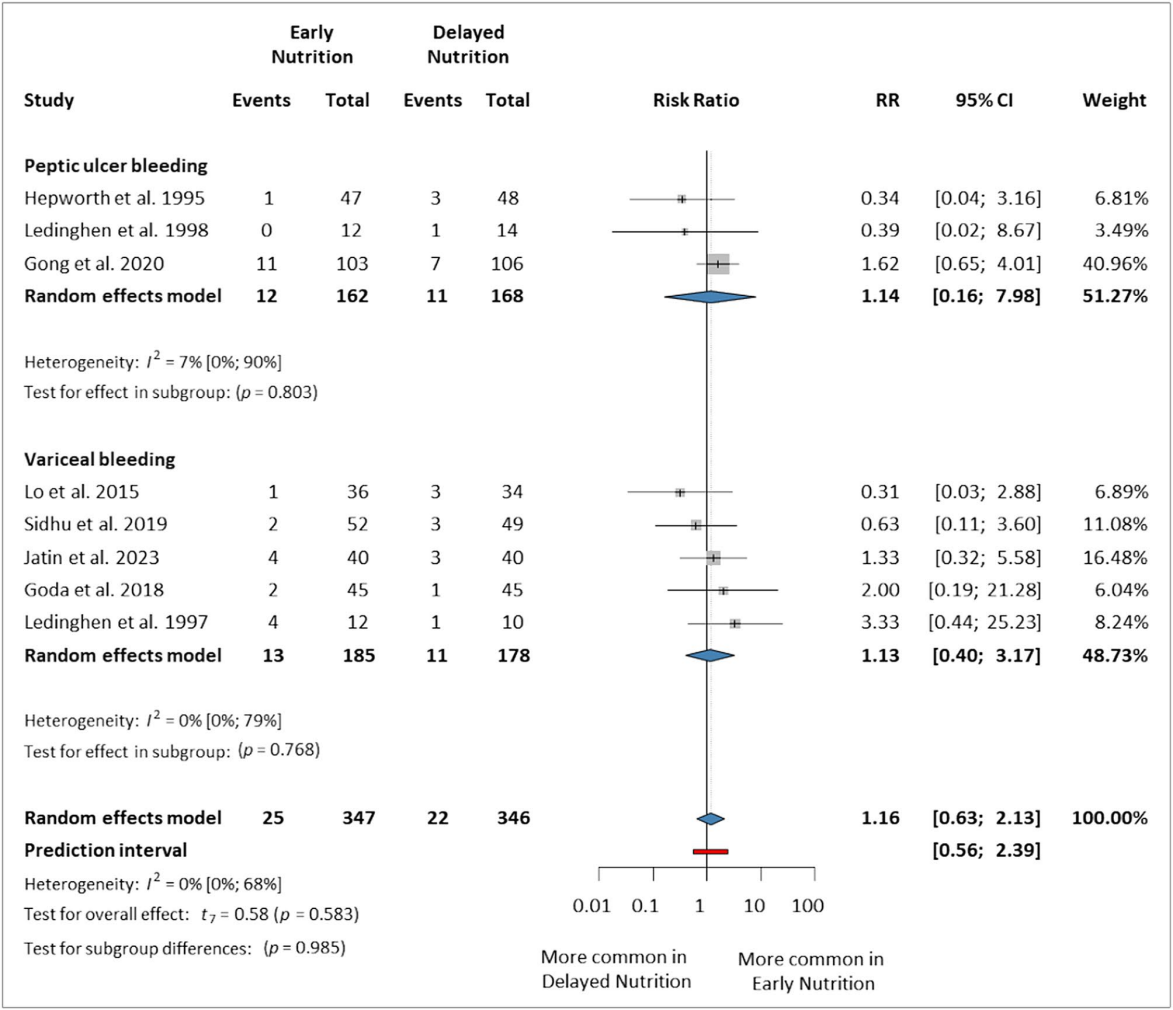
Forest plot demonstrating the effect of early versus delayed nutrition on late rebleeding (within 30–42 days) after upper gastrointestinal bleeding. *RR* risk ratio, *CI* confidence interval.

This analysis included eight studies^5,7,14–16,44,46,47^ that reported rebleeding within 30, 35, or 42 days, involving 693 patients (347 in the EN group and 346 in the DN group). The results were not statistically significant for either subgroup, including PUB (RR 1.14, 95% CI 0.16–7.98) and VUGIB (RR 1.13, 95% CI 0.40–3.17). Overall, EN did not increase the risk of late rebleeding compared to DN (RR 1.16, 95% CI 0.63–2.13, *p* = 0.583, *I*^2^ = 0%, 95% CI 0–68%) (Fig. 3).

### Early mortality (within 7 days)

**Figure 4:**
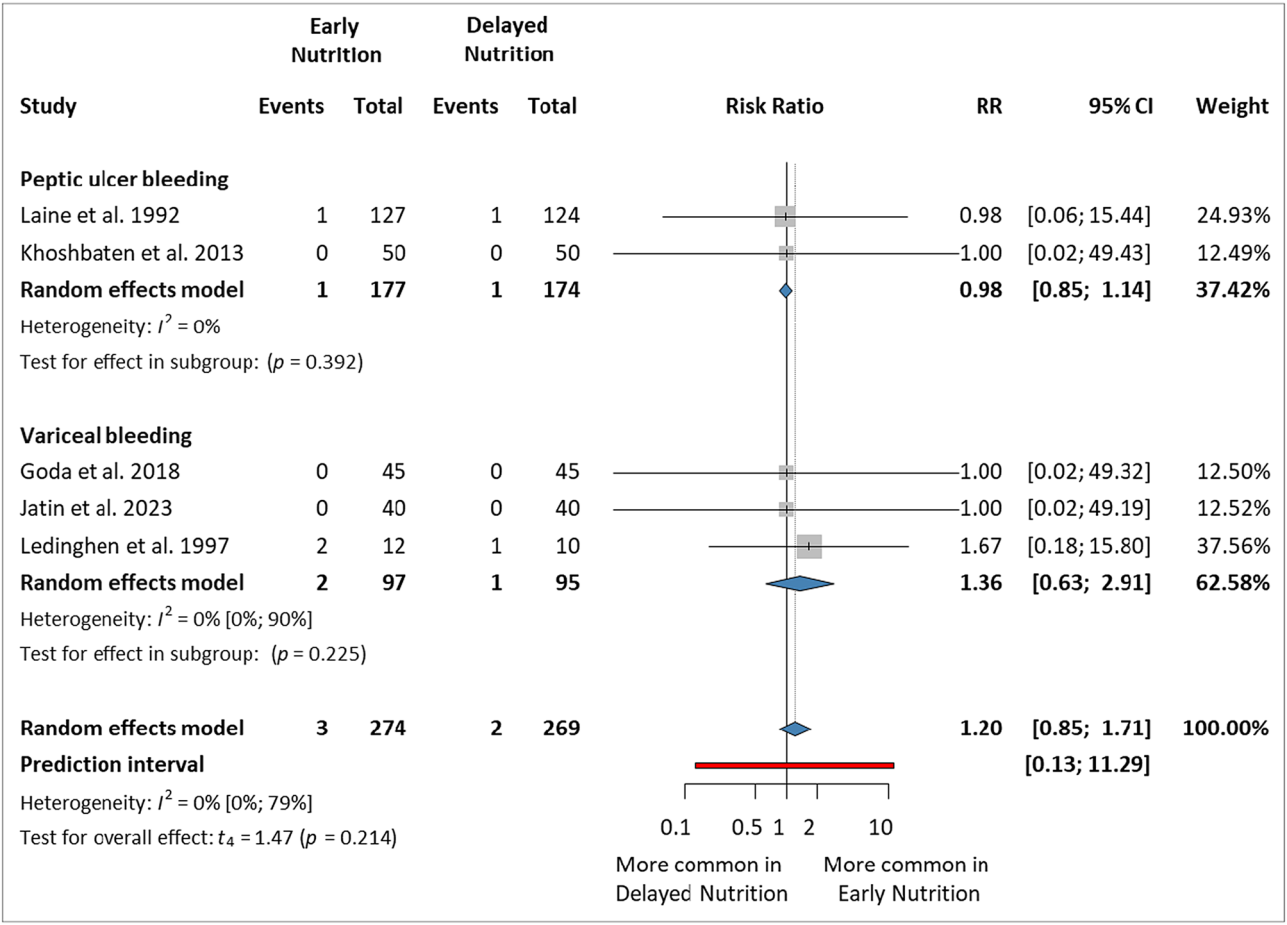
Forest plot demonstrating the effect of early versus delayed nutrition on early mortality (within 7 days) after upper gastrointestinal bleeding. *RR* risk ratio, *CI* confidence interval.

Only five studies^7,13,15,44,45^ reporting mortality within seven days were included in this analysis, with a total of 543 patients (234 in the EN group and 229 in the DN group). There were no statistically significant differences between the studies in the PUB and VUGIB subgroups (RR 0.98, 95% CI 0.85–1.14, and RR 1.36, 95% CI 0.63–2.91, respectively). The overall effect was not statistically significant between the two groups (RR 1.20, 95% CI 0.85–1.71, *p* = 0.214, *I*^2^ = 0%, 95% CI 0–79%) (Fig. 4).

### Late mortality (within 30–42 days)

**Figure 5:**
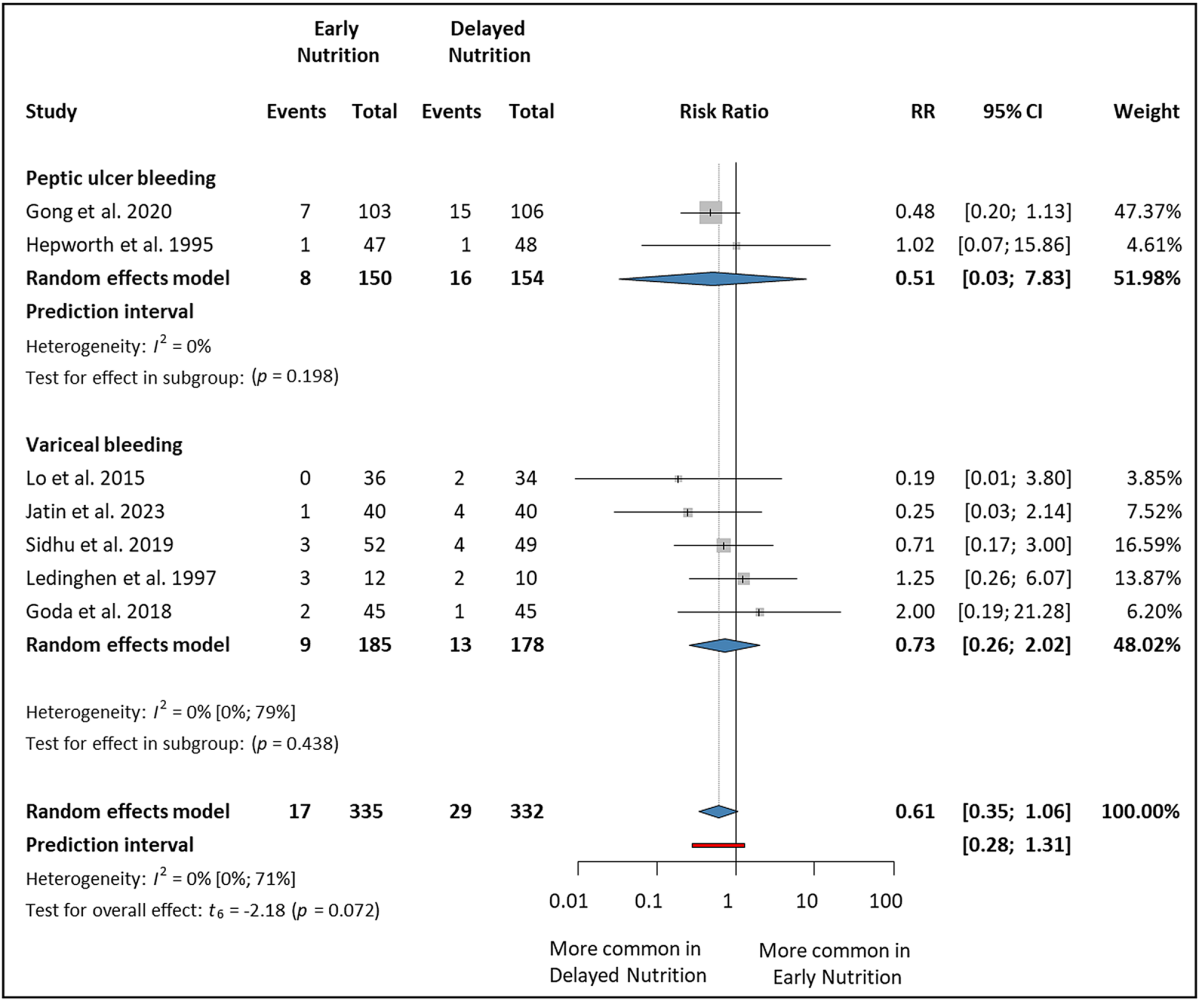
Forest plot demonstrating the effect of early versus delayed nutrition on late mortality (within 30–42 days) after upper gastrointestinal bleeding. *RR* risk ratio, *CI* confidence interval.

The analysis included seven studies^5,7,14–16,44,47^ that reported mortality within 30, 35, or 42 days. Altogether, 667 patients were involved (335 in the EN group and 332 in the DN group). There was no statistical difference in either subgroup; in the PUB subgroup (RR 0.51, 95% CI 0.03–7.83) and the VUGIB subgroup (RR 0.73, 95% CI 0.26–2.02). Overall, there was no statistically significant difference between the two groups; however, the results were clinically relevant with a tendency towards the EN group (RR 0.61, 95% CI 0.35–1.06, *p* = 0.072, *I*^2^ = 0%, 95% CI 0–71%) (Fig. 5).

### Length of hospital stay (days)

**Figure 6:**
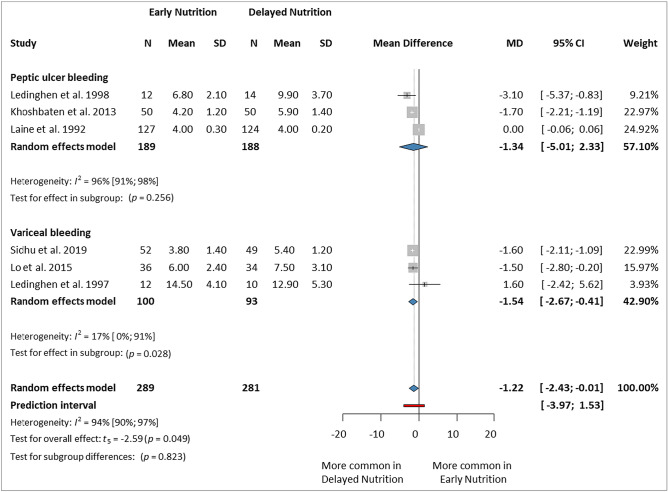
Forest plot demonstrating the effect of early versus delayed nutrition on the length of hospital stay after upper gastrointestinal bleeding. *N* number of patients in each arm, *SD* standard deviation, *MD* mean difference, *CI* confidence interval.

This analysis included six studies^5,7,13,14,45,46^ involving 570 patients (289 in the EN group and 281 in the DN group). In the PUB subgroup, there was no statistically significant difference between the two groups (MD: −1.34 days, 95% CI − 5.01 to 2.33), whereas in the VUGIB subgroup, EN significantly decreased LOS (MD −1.54 days, 95% CI −2.67 to −0.41). Overall, EN reduced the LOS compared to DN (MD −1.22 days, 95% CI −2.43 to −0.01, *p* = 0.049, *I*^2^ = 94%, 95% CI 90–97%) (Fig. 6).

Jatin et al.^15^ and Gong et al.^16^ reported their results in medians instead of means. We decided to exclude those studies from our final analysis due to potentially biased results. However, even if we estimated the means from the provided medians, the findings indicate a significant reduction in LOS (MD −1.12 days, 95% CI −2.03 to −0.22, *p* = 0.022, *I*^2^ = 93%, 95% CI 88–96%) (Supplementary Material Fig. S1).

### Blood transfusion requirement (units)

Seven studies^5,7,13,14,16,45,46^ reported transfusion requirement as an outcome; however, we could analyze only four studies (292 in the EN group and 286 in the DN group)^5,16,45,46^ due to heterogeneous definitions of this outcome among the included studies (summarized in the Supplementary Material Table S3). Overall, there was no statistically significant difference between the two groups (MD 0.00, 95% CI −0.04 to 0.05, *p* = 0.980, *I*^2^ = 0%, 95% CI 0–85%). (Supplementary Material Fig. S2).

### Bacterial infection

Only three studies^5,14,15^ reported new-onset bacterial infections, including 251 patients (128 in the EN group and 123 in the DN group). Overall, there was no statistically significant difference between the two groups (RR 0.48, 95% CI 0.08–3.05, *p* = 0.229, *I*^2^ = 0%, 95% CI 0–90%) (Supplementary Material Fig. S3).

### Ascites and hepatic encephalopathy

Three studies reported on new-onset ascites^5,14,15^. Overall, there was a tendency that ascites was more common in the DN group, however, it was not statistically significant (RR 0.64, 95% CI 0.34–1.20, *p* = 0.094, *I*^2^ = 0%, 95% CI 0–90%) (Supplementary Material Fig. S4). In addition, two studies^5,15^ reported on new-onset hepatic encephalopathy. (RR 1.03, 95% CI 0.50–2.11 and RR 0.75, 95% CI 0.18–3.14, respectively). We were not able to draw a statistical inference based on only two studies; therefore, we presented this outcome in the Supplementary Material Fig. S5 without an overall effect.

### ICU admission and transfusion rate

We were not able to come to any statistical conclusions regarding these outcomes. Only one study^45^ reported the ICU admission days; there was no difference between the two groups (EN: 1.1 ± 0.2, DN: 1.1 ± 0.1), and one study^15^ reported the need for ICU admission (EN: 1/40, DN: 2/40). No studies reported data on the transfusion rate.

### Risk of bias assessment and quality of evidence

In the articles, the randomization process and the selection of the reported result domains raised "some concerns". Deviations from the planned intervention and missing outcome data had the lowest risk of bias. Bias from outcome measurement was high in LOS and new-onset hepatic encephalopathy outcomes. Results of risk of bias assessment for all the included studies by outcome are presented in the Supplementary Material Figs. S6–S12.

The quality of evidence was low or very low for all our outcomes. A summary table and explanation of the results can be found in the Supplementary Material (Supplementary Tables S4–S5).

### Heterogeneity and publication bias

All analyses of the outcomes included showed negligible statistical heterogeneity levels (*I*^2^), with heterogeneity of 0% or less than 10%, except for the LOS outcome, which was 94% (CI 90–97%). This discrepancy may be attributed to variations in bleeding severity among patients, impacting hospitalization needs. We realized that when omitting Laine et al. study^45^ with leave-one-out sensitivity analysis, which included less severe patients compared to other studies, the effect size became more significant (MD −1.65 days, 95% CI −1.99 to − 1.31), and heterogeneity decreased to 5% (Supplementary Fig. S13–14). As for publication bias, the test's diagnostic accuracy is limited for studies below 10, and as none of our analyses met this threshold, we opted to exclude this analysis.

## Discussion

Our study found no significant difference in early and late rebleeding and mortality between EN and DN after UGIB hemostasis; however, these findings are clinically relevant. The results showed that EN could significantly decrease LOS compared to DN. In addition, there was no difference between the two groups in terms of blood transfusion requirement and bacterial infection (Supplementary Material Fig. S15).

Despite the advances in intensive care technologies and improvements in the endoscopic treatment of GIB, it remains a life-threatening emergency with considerable mortality^1^. Our study revealed a late mortality ratio of 6.89% (46 out of 667 cases), indicating an important concern; we reached a similar conclusion in terms of late rebleeding, with a ratio of 6.78% (47 out of 693 cases).

The precise definitions of both interventions (early and delayed nutrition) played an essential role in determining the timing, dietary type, procedure, and requirements, particularly because there were variations in how these interventions were defined across the included studies. To address this, we have compiled a comprehensive overview of the definitions for both interventions, which can be found in the Supplementary Material Table S6.

According to the current literature, patients at a high risk of rebleeding should be advised to fast and remain hospitalized for a minimum of 48–72 h after endoscopic treatment. Within this timeframe, most high-risk lesions will transition into low-risk lesions, and most rebleeding events will occur^8^. Therefore, prolonged fasting can be justified. Furthermore, a retrospective study^48^ showed that delaying refeeding in patients with low-risk lesions who should have been fed promptly is not advisable, and early refeeding is recommended for NVUGIB patients. According to a recent review^11^, the timing of initiating feeding after the diagnosis of UGIB should be determined by considering patient-specific risk factors associated with the underlying disease. For low-risk patients, it is advisable to resume feeding without delay following endoscopy, as these bleeds are often self-limited and rarely require intervention. However, for higher-risk lesions (Forrest Ia–IIb), the available data on the safety of early refeeding are inconclusive^11^. This was also a challenge in our analysis, as different studies included patients with varying severities. For example, Gong et al.^16^ included FIa–FIb bleeders, whereas Khoshbaten et al.^13^ did not.

Enteral nutrition has the potential to provide several benefits. These include the delivery of local nutrition directly to the gastric tissue, stimulating mucus glands and epithelial cells to support the maintenance of the protective mucus barrier, and promoting increased blood flow to the splanchnic region, which can aid ulcer healing^8^. In addition, a prospective study^49^ aimed to compare the early and late postoperative oral feeding of gastric cancer patients undergoing surgery for the recovery of gastrointestinal function. It was found that initiating early oral feeding in patients with gastric cancer facilitates the recovery of postoperative gastrointestinal function without increasing the rate of associated complications or adverse events. Another meta-analysis^50^ concluded that, in comparison to traditional oral feeding, early refeeding after upper gastrointestinal surgery could shorten the LOS and time of first exhaust without increasing postoperative complications, while also reducing the risk of pneumonia.

Several reviews^51–53^ suggest that enteral nutrition may protect against stress ulceration. Numerous studies in basic science indicate that enteral nutrition can enhance mucosal blood flow and reverse the production of inflammatory mediators^53^. In addition, the results of a meta-analysis^54^ indicated that stress ulcer prophylaxis with a histamine-2 receptor blocker may not be necessary for patients receiving enteral nutrition. They found that prophylactic use of a histamine-2 receptor blocker for stress ulcer prevention resulted in a decreased risk of GIB with an odds ratio (OR) of 0.47 (95% CI 0.29–0.76; *p* < 0.002) However, this treatment effect was observed only in patients who did not receive enteral nutrition. Among patients who were fed enterally, stress ulcer prophylaxis did not have a significant impact on the risk of GIB (OR 1.26; 95% CI 0.43–3.7).

In contrast to the meta-analysis by Zhang et al.^17^, our study extended their research by including five more clinical trials and examining a broader range of outcomes. Furthermore, we conducted a subgroup analysis based on the source of bleeding, which allowed for more accurate and specific data in our investigation. Their findings also suggested that EN administered within 24 h did not show a higher risk of rebleeding and mortality compared to DN for patients with UGIB. However, EN was associated with a reduction in the LOS.

Risk-stratification systems have been developed to differentiate between patients with a high or low risk of mortality or rebleeding in cases of GIB. However, many of these scores rely on endoscopic findings, which makes them less suitable for early patient evaluation. Fortunately, several risk scores, such as the AIMS65 and Glasgow–Blatchford scores, can be used prior to endoscopy. Therefore, it is crucial to introduce these risk-stratification systems into clinical practice and apply them to determine the optimal timing for initiating enteral nutrition^55–57^.

Regarding the strengths of our analysis, we strictly adhered to our protocol, which was registered beforehand. Our study is the most recent comprehensive analysis of refeeding strategies after UGIB using a rigorous methodology and including only RCTs. In addition, we performed a subgroup analysis based on bleeding source, providing more detailed data.

As for the limitations of this work, only a few studies with a low number of cases could be included. In addition, the EN and DN definitions varied among the studies, and different nutrition modalities and regimens were used. Generalizing the findings might be challenging due to variations in the severity of bleeding among the included patients, which could impact the appropriate timing for refeeding. Other limitations include a high risk of bias in some of the domains and the low quality of evidence.

Our results suggest that EN is a safe intervention; however, further high-quality prospective data collection and reporting are needed to assess this clinical question more accurately, including clinical trials reporting the investigated outcomes based on the severity assessment with longer follow-up periods, others on the diet types and their effects on new-onset ascites, and hepatic encephalopathy might give additional insight into this field. In addition, the emphasis on adherence to risk stratification scores prior to endoscopy ensures appropriate management of those patients^58,59^.

## Conclusion

In comparison to delayed nutrition, early nutrition (within 24 h) appears to be a safe intervention and could reduce the length of hospital stay without increasing the risk of complications in terms of rebleeding or mortality after hemostasis of upper gastrointestinal bleeding.

### Supplementary Information

Supplementary Information.

## Data Availability

All the data analyzed in this study are available in the full text of the included studies and supplementary material.
