# Supplementary material for: Early nutrition is safe and does not increase complications after upper gastrointestinal bleeding—a systematic review and meta-analysis of randomized controlled trials
Source: Sci Rep. 2024 May 10;14:10725. doi: 10.1038/s41598-024-61543-z (PMC11087503; doi:10.1038/s41598-024-61543-z)
Supplement: Supplementary file 1 — Supplementary Information. [file 41598_2024_61543_MOESM1_ESM.docx]

**SUPPLEMENTARY MATERIAL**

**TITLE**

Early nutrition is safe and does not increase complications after upper gastrointestinal bleeding – a systematic review and meta-analysis of randomized controlled trials

**AUTHORS**

Mahmoud Obeidat^1,2^, Brigitta Teutsch^1,2^, Diana-Elena Floria^1,3^, Dániel Sándor Veres^1,4^, Péter Hegyi^1,2,5^, Bálint Erőss*^1,2,5^

**AFFILIATIONS**

1. Centre for Translational Medicine, Semmelweis University, Budapest 1085, Hungary
2. Institute for Translational Medicine, Medical School, University of Pécs, Pécs 7623, Hungary
3. Grigore T. Popa University of Medicine and Pharmacy, Iași 700115, Romania
4. Department of Biophysics and Radiation Biology, Semmelweis University, Budapest 1085, Hungary
5. Institute of Pancreatic Diseases, Semmelweis University, Budapest 1083, Hungary

**CORRESPONDING AUTHOR**

Bálint Erőss MD, PhD, FRCP (London)

Semmelweis University, Centre or Translational Medicine, Üllői út 26, Budapest, H-1085, Hungary

E-mail address: [dr.eross.balint@gmail.com](mailto:dr.eross.balint@gmail.com)

**Figure Legends**

**Figure S1.** Forest plot demonstrating the effect of early versus delayed nutrition on the length of hospital stay (including Gong et al. and Jatin et al. studies) after upper gastrointestinal bleeding. N, number of patients in each arm; SD, standard deviation; MD, mean difference; CI, confidence interval.

**Figure S2.** Forest plot demonstrating the effect of early versus delayed nutrition on blood transfusion requirements after upper gastrointestinal bleeding. N, number of patients in each arm; SD, standard deviation; MD, mean difference; CI, confidence interval.

**Figure S3.** Forest plot demonstrating the effect of early versus delayed nutrition on the incidence of bacterial infection after upper gastrointestinal bleeding. RR, risk ratio; CI, confidence interval.

**Figure S4.** Forest plot demonstrating the effect of early versus delayed nutrition on the incidence of newly developed ascites after upper gastrointestinal bleeding. RR, risk ratio; CI, confidence interval.

**Figure S5.** Forest plot demonstrating the effect of early versus delayed nutrition on the incidence of newly developed hepatic encephalopathy after upper gastrointestinal bleeding. RR, risk ratio; CI, confidence interval.

**Figure S6.** The risk of bias assessment at the study and domain level for the rebleeding rate.

**Figure S7.** The risk of bias assessment at the study and domain level for the mortality rate.

**Figure S8.** The risk of bias assessment at the study and domain level for the length of hospital stay.

**Figure S9.** The risk of bias assessment at the study and domain level for the incidence of bacterial infection.

**Figure S10.** The risk of bias assessment at the study and domain level for the blood transfusion requirement.

**Figure S11.** The risk of bias assessment at the study and domain level for the newly developed ascites.

**Figure S12.** The risk of bias assessment at the study and domain level for the newly developed hepatic encephalopathy.

**Figure S13.** Leave-one-out sensitivity analysis in the length of hospital stay days based on the effect size. MD, mean difference; CI, confidence interval.

**Figure S14.** Leave-one-out sensitivity analysis in the length of hospital stay days sorted by the heterogeneity level.

**Figure S15.** Summary forest plots demonstrating the effect of early versus delayed nutrition in all the investigated outcomes. **A**. Binary outcomes. RR, risk ratio; CI, confidence interval. **B**. Continuous outcomes. MD, mean difference; CI, confidence interval**.**

**Table Legends**

**Table S1.** PRISMA checklist.

**Table S2.** The detailed search key.

**Table S3**. Summary table of studies reporting blood transfusion requirements and their definitions.

**Table S4.** Summary of findings table of the quality of evidence of the rebleeding, mortality and length of hospital stay.

**Table S5.** Summary of findings table of the quality of evidence of the blood transfusion requirement, bacterial infection, and new-onset ascites outcomes.

**Table S6.** Detailed definitions of the timing and dietary nutrition in the included studies.

**Table S1. PRISMA checklist**

| **Section and Topic** | **Item #** | **Checklist item** | **Location where item is reported** |
| --- | --- | --- | --- |
| **TITLE** | | |  |
| Title | 1 | Identify the report as a systematic review. | 1 |
| **ABSTRACT** | | |  |
| Abstract | 2 | See the PRISMA 2020 for Abstracts checklist. | 2 |
| **INTRODUCTION** | | |  |
| Rationale | 3 | Describe the rationale for the review in the context of existing knowledge. | 3-4 |
| Objectives | 4 | Provide an explicit statement of the objective(s) or question(s) the review addresses. | 3-4 |
| **METHODS** | | |  |
| Eligibility criteria | 5 | Specify the inclusion and exclusion criteria for the review and how studies were grouped for the syntheses. | 4 |
| Information sources | 6 | Specify all databases, registers, websites, organisations, reference lists and other sources searched or consulted to identify studies. Specify the date when each source was last searched or consulted. | 4 |
| Search strategy | 7 | Present the full search strategies for all databases, registers and websites, including any filters and limits used. | 5 and Supplementary Table S2 |
| Selection process | 8 | Specify the methods used to decide whether a study met the inclusion criteria of the review, including how many reviewers screened each record and each report retrieved, whether they worked independently, and if applicable, details of automation tools used in the process. | 5 |
| Data collection process | 9 | Specify the methods used to collect data from reports, including how many reviewers collected data from each report, whether they worked independently, any processes for obtaining or confirming data from study investigators, and if applicable, details of automation tools used in the process. | 5 |
| Data items | 10a | List and define all outcomes for which data were sought. Specify whether all results that were compatible with each outcome domain in each study were sought (e.g. for all measures, time points, analyses), and if not, the methods used to decide which results to collect. | 5 |
|  | 10b | List and define all other variables for which data were sought (e.g. participant and intervention characteristics, funding sources). Describe any assumptions made about any missing or unclear information. | 5 and Table 1 and Supplementary Table S6 |
| Study risk of bias assessment | 11 | Specify the methods used to assess risk of bias in the included studies, including details of the tool(s) used, how many reviewers assessed each study and whether they worked independently, and if applicable, details of automation tools used in the process. | 6 |
| Effect measures | 12 | Specify for each outcome the effect measure(s) (e.g. risk ratio, mean difference) used in the synthesis or presentation of results. | 6-7 |
| Synthesis methods | 13a | Describe the processes used to decide which studies were eligible for each synthesis (e.g. tabulating the study intervention characteristics and comparing against the planned groups for each synthesis (item #5)). | 6-7 |
|  | 13b | Describe any methods required to prepare the data for presentation or synthesis, such as handling of missing summary statistics, or data conversions. | 6-7 |
|  | 13c | Describe any methods used to tabulate or visually display results of individual studies and syntheses. | 6-7 |
|  | 13d | Describe any methods used to synthesize results and provide a rationale for the choice(s). If meta-analysis was performed, describe the model(s), method(s) to identify the presence and extent of statistical heterogeneity, and software package(s) used. | 6-7 |
|  | 13e | Describe any methods used to explore possible causes of heterogeneity among study results (e.g. subgroup analysis, meta-regression). | 6-7 |
|  | 13f | Describe any sensitivity analyses conducted to assess robustness of the synthesized results. | 6-7 |
| Reporting bias assessment | 14 | Describe any methods used to assess risk of bias due to missing results in a synthesis (arising from reporting biases). | 6-7 |
| Certainty assessment | 15 | Describe any methods used to assess certainty (or confidence) in the body of evidence for an outcome. | 6 |
| **RESULTS** | | |  |
| Study selection | 16a | Describe the results of the search and selection process, from the number of records identified in the search to the number of studies included in the review, ideally using a flow diagram. | 8 (Figure 1) |
|  | 16b | Cite studies that might appear to meet the inclusion criteria, but which were excluded, and explain why they were excluded. | 8 |
| Study characteristics | 17 | Cite each included study and present its characteristics. | 9 and Table 1 |
| Risk of bias in studies | 18 | Present assessments of risk of bias for each included study. | Supplementary Figures S6-S12 |
| Results of individual studies | 19 | For all outcomes, present, for each study: (a) summary statistics for each group (where appropriate) and (b) an effect estimate and its precision (e.g. confidence/credible interval), ideally using structured tables or plots. | Figures 2-6 and supplementary Figures S1-S5 |
| Results of syntheses | 20a | For each synthesis, briefly summarise the characteristics and risk of bias among contributing studies. | Table 1 and Supplementary Figures S6-S12 |
|  | 20b | Present results of all statistical syntheses conducted. If meta-analysis was done, present for each the summary estimate and its precision (e.g. confidence/credible interval) and measures of statistical heterogeneity. If comparing groups, describe the direction of the effect. | Figures 2-6 and supplementary Figures S1-S5 |
|  | 20c | Present results of all investigations of possible causes of heterogeneity among study results. | - |
|  | 20d | Present results of all sensitivity analyses conducted to assess the robustness of the synthesized results. | Supplementary Figure S13-14 |
| Reporting biases | 21 | Present assessments of risk of bias due to missing results (arising from reporting biases) for each synthesis assessed. | - |
| Certainty of evidence | 22 | Present assessments of certainty (or confidence) in the body of evidence for each outcome assessed. | Supplementary Table S4-S5 |
| **DISCUSSION** | | |  |
| Discussion | 23a | Provide a general interpretation of the results in the context of other evidence. | 17 |
|  | 23b | Discuss any limitations of the evidence included in the review. | 19 |
|  | 23c | Discuss any limitations of the review processes used. | 19 |
|  | 23d | Discuss implications of the results for practice, policy, and future research. | 20 |
| **OTHER INFORMATION** | | |  |
| Registration and protocol | 24a | Provide registration information for the review, including register name and registration number, or state that the review was not registered. | 4 |
|  | 24b | Indicate where the review protocol can be accessed, or state that a protocol was not prepared. | 4 |
|  | 24c | Describe and explain any amendments to information provided at registration or in the protocol. | 4 |
| Support | 25 | Describe sources of financial or non-financial support for the review, and the role of the funders or sponsors in the review. | 24 |
| Competing interests | 26 | Declare any competing interests of review authors. | 24 |
| Availability of data, code and other materials | 27 | Report which of the following are publicly available and where they can be found: template data collection forms; data extracted from included studies; data used for all analyses; analytic code; any other materials used in the review. | 20 |

| **Table S2. The detailed search key** | |
| --- | --- |
| **Database** | **Search Key** |
| **Embase** | (((oral or enteral or early or immediate* or delayed or late) and (feeding OR nutrition OR refeeding)) OR enteral nutrition OR enteric feeding) AND (gastrointestinal haemorrhage OR gastrointestinal hemorrhage OR gastrointestinal bleed* OR GI bleed* OR GIB OR UGIB OR ((nonvariceal OR non variceal OR non-variceal OR variceal OR varix OR ulcer) AND (bleeding or hemorrhage or haemorrhage))) AND random* |
| **CENTRAL** | (((oral or enteral or early or immediate* or delayed or late) and (feeding OR nutrition OR refeeding)) OR enteral nutrition OR enteric feeding) AND (gastrointestinal haemorrhage OR gastrointestinal hemorrhage OR gastrointestinal bleed* OR GI bleed* OR GIB OR UGIB OR ((nonvariceal OR non variceal OR non-variceal OR variceal OR varix OR ulcer) AND (bleeding or hemorrhage or haemorrhage))) AND random* |
| **PubMed** | (((oral or enteral or early or immediate* or delayed or late) and (feeding OR nutrition OR refeeding)) OR enteral nutrition OR enteric feeding) AND (gastrointestinal haemorrhage OR gastrointestinal hemorrhage OR gastrointestinal bleed* OR GI bleed* OR GIB OR UGIB OR ((nonvariceal OR non variceal OR non-variceal OR variceal OR varix OR ulcer) AND (bleeding or hemorrhage or haemorrhage))) AND random* |
| **Scopus** | (((oral or enteral or early or immediate* or delayed or late) and (feeding OR nutrition OR refeeding)) OR enteral nutrition OR enteric feeding) AND (gastrointestinal haemorrhage OR gastrointestinal hemorrhage OR gastrointestinal bleed* OR GI bleed* OR GIB OR UGIB OR ((nonvariceal OR non variceal OR non-variceal OR variceal OR varix OR ulcer) AND (bleeding or hemorrhage or haemorrhage))) AND random* |
| **Web of Science** | (((oral or enteral or early or immediate* or delayed or late) and (feeding OR nutrition OR refeeding)) OR enteral nutrition OR enteric feeding) AND (gastrointestinal haemorrhage OR gastrointestinal hemorrhage OR gastrointestinal bleed* OR GI bleed* OR GIB OR UGIB OR ((nonvariceal OR non variceal OR non-variceal OR variceal OR varix OR ulcer) AND (bleeding or hemorrhage or haemorrhage))) AND random* |


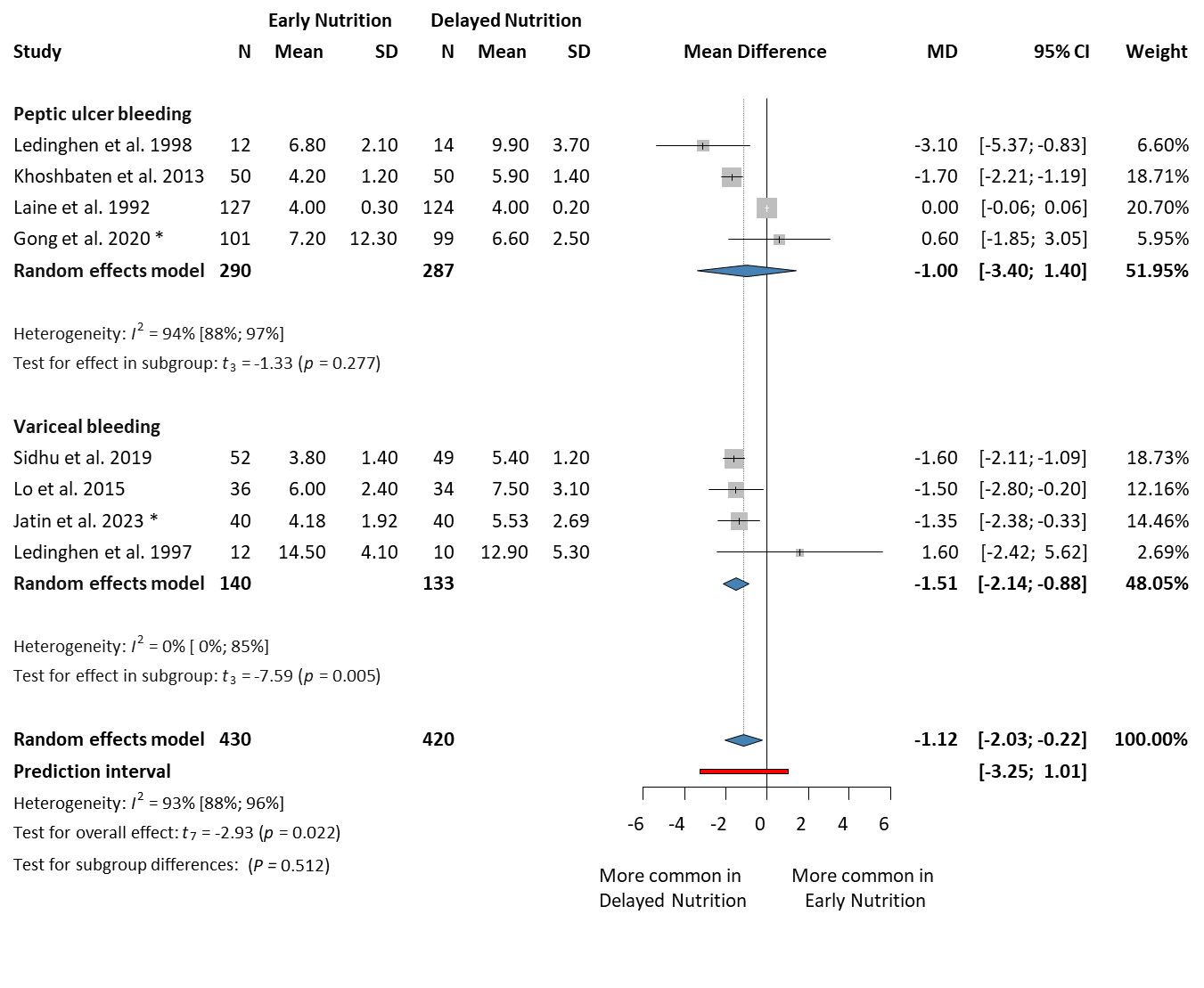


**Figure S1**. Forest plot demonstrating the effect of early versus delayed nutrition on the length of hospital stay (including Gong et al. and Jatin et al. studies) after upper gastrointestinal bleeding. N, number of patients in each arm; SD, standard deviation; MD, mean difference; CI, confidence interval

* Mean is estimated from the median

| **Table S3.** Summary table of studies reporting blood transfusion requirement and definitions | | | | | | | |
| --- | --- | --- | --- | --- | --- | --- | --- |
| **Study** | **Early Nutrition** | | | **Delayed Nutrition** | | | **Definition of blood transfusion  (mean number of units)** |
|  | **Total** | **Mean** | **SD** | **Total** | **Mean** | **SD** |  |
| Gin-Ho Lo et al. 2015 ^1^ | 36 | 3.10 | 2.30 | 34 | 2.70 | 2.2 | At baseline |
|  | 36 | 1.83 | 1.25 | 34 | 2.15 | 1.6 | After endoscopy |
| Khoshbaten et al. 2013 ^2^ | 50 | 0.80 | 0.50 | 50 | 1.40 | 0.70 | Before intervention |
|  | 50 | 1.20 | 0.60 | 50 | 1.60 | 0.70 | After intervention |
| Ledinghen et al. 1997 ^3^ | 12 | 2.00 | 1.30 | 10 | 1.80 | 1.50 | Within the first 24h |
|  | 12 | 1.60 | 0.50 | 10 | 1.00 | 0.40 | From day 1 through day 7 |
| Laine et al. 1992 ^4^ | 127 | 0.80 | 0.20 | 124 | 0.80 | 0.10 | After randomization |
| Ledinghen et al. 1998 ^5^ | 12 | 2.60 | 2.10 | 14 | 3.30 | 2.10 | Transfusion requirement |
| Sidhu et al. 2019 ^6^ | 52 | 3.10 | 2.30 | 49 | 2.70 | 2.20 | Blood unit transfused |
| Gong el at. 2020* ^7^ | 101 | 2.20 | 1.70 | 99 | 2.20 | 1.80 | The median number of transfused units |
| * Gong et al. 2020 study reported the median value of transfused blood units | | | | | | | |

**
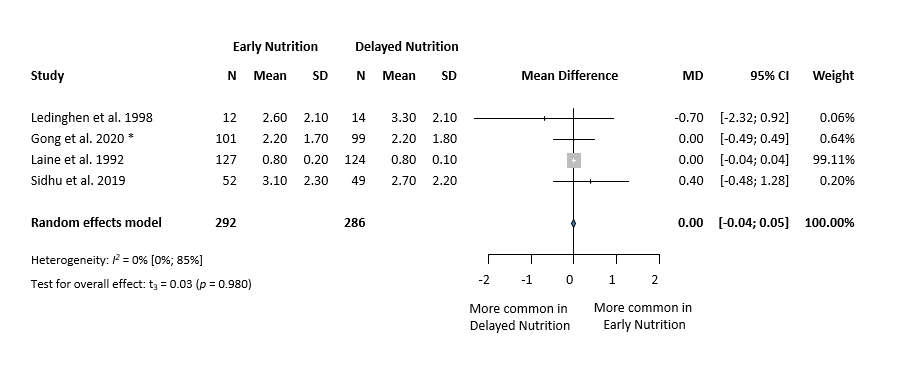
**

**Figure S2.** Forest plot demonstrating the effect of early versus delayed nutrition on blood transfusion requirements after upper gastrointestinal bleeding. N, number of patients in each arm; SD, standard deviation; MD, mean difference; CI, confidence interval

* Mean is estimated from the median in Gong et al. study

**
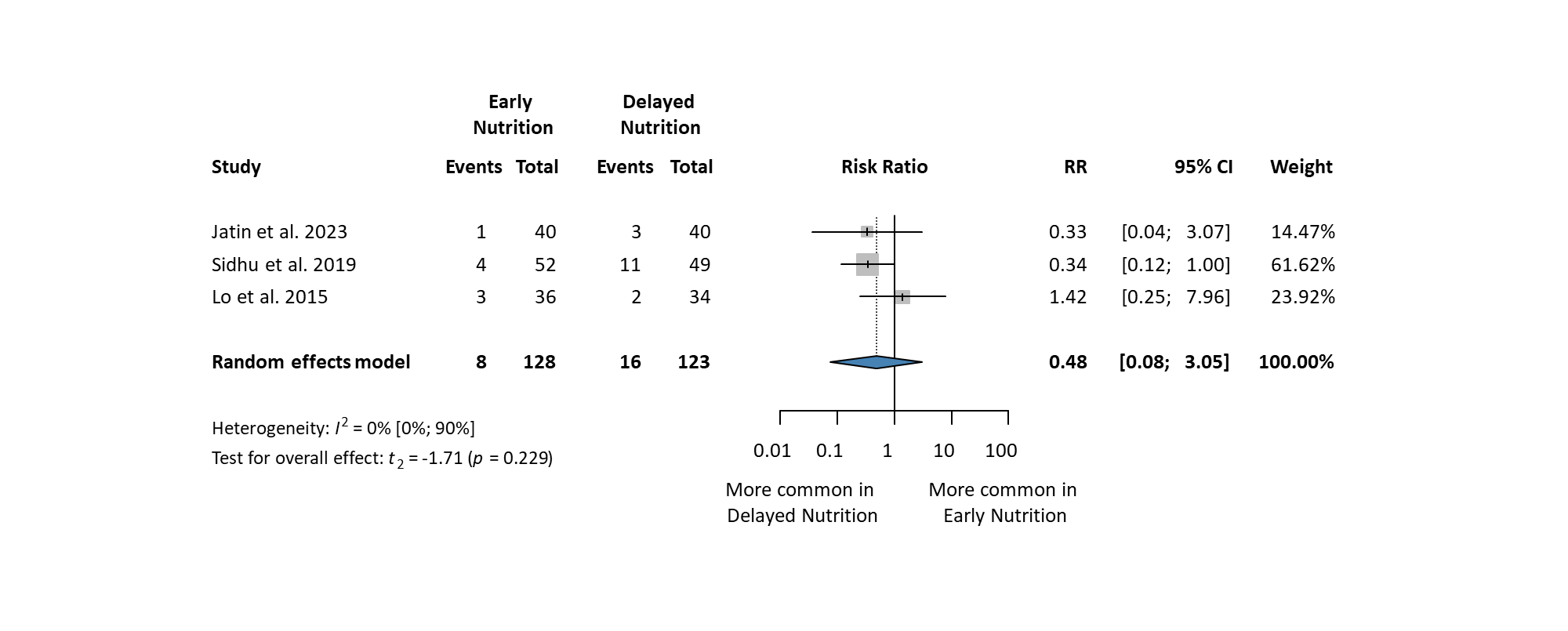
**

**Figure S3.** Forest plot demonstrating the effect of early versus delayed nutrition on the incidence of bacterial infection after upper gastrointestinal bleeding. RR, risk ratio; CI, confidence interval.

**
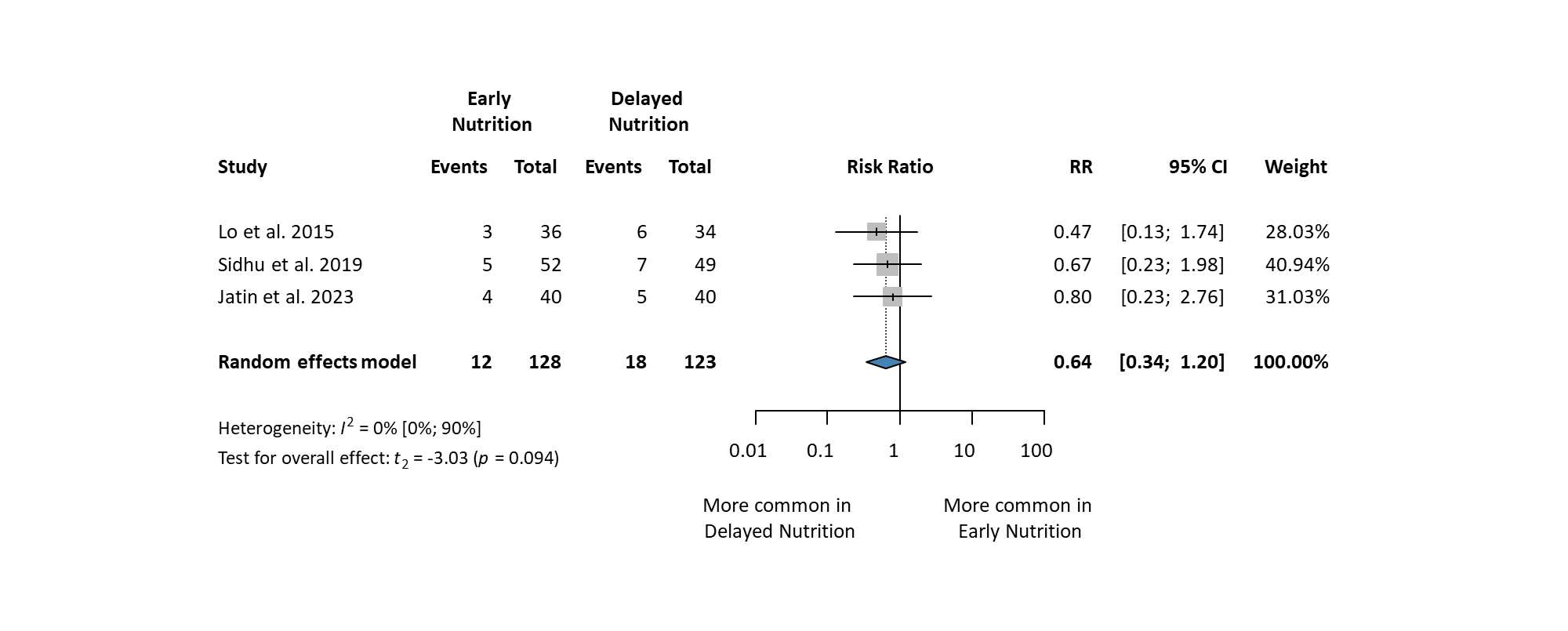
**

**
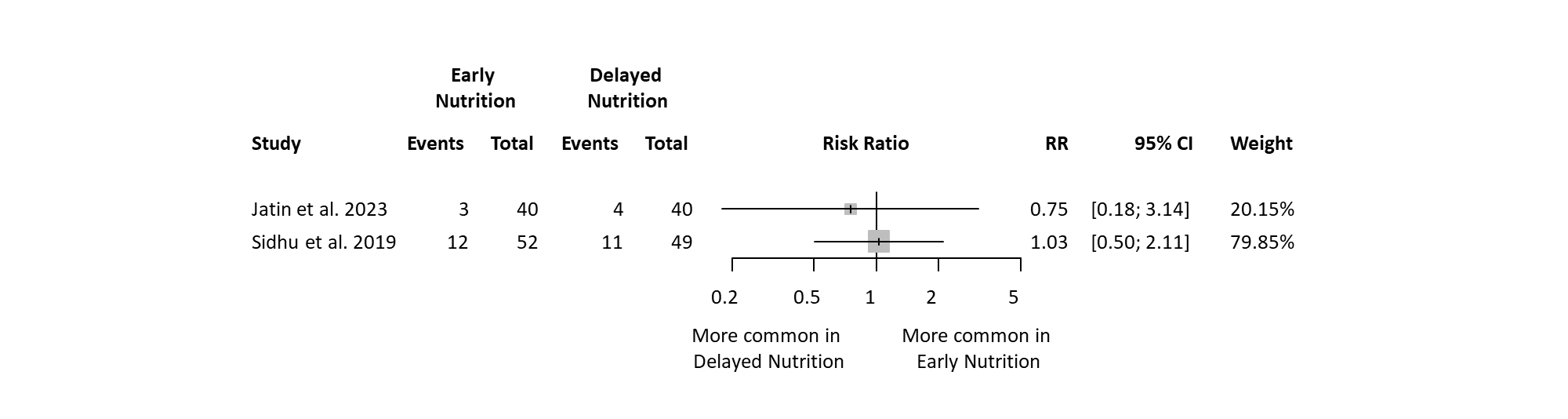
**

**Figure S4**. Forest plot demonstrating the effect of early versus delayed nutrition on the incidence of newly developed ascites after upper gastrointestinal bleeding. RR, risk ratio; CI, confidence interval.

**Figure S5.** Forest plot demonstrating the effect of early versus delayed nutrition on the incidence of newly developed hepatic encephalopathy after upper gastrointestinal bleeding. RR, risk ratio; CI, confidence interval.

| 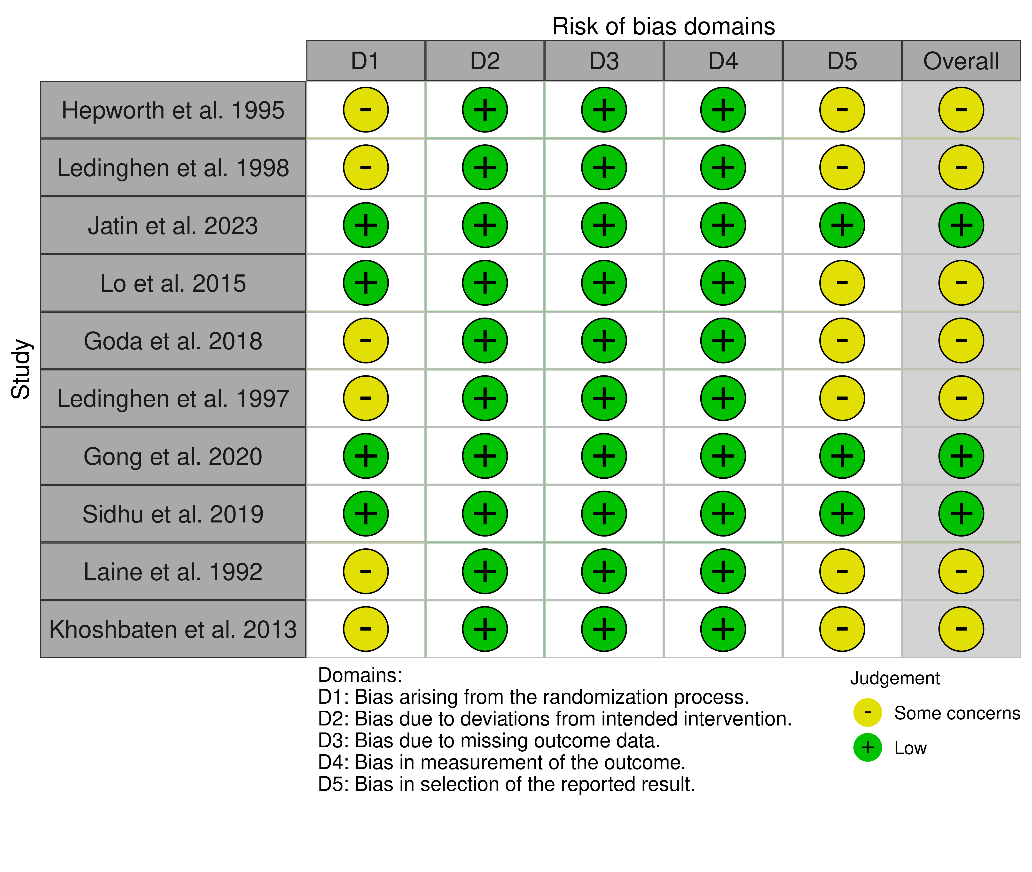 |
| --- |
| 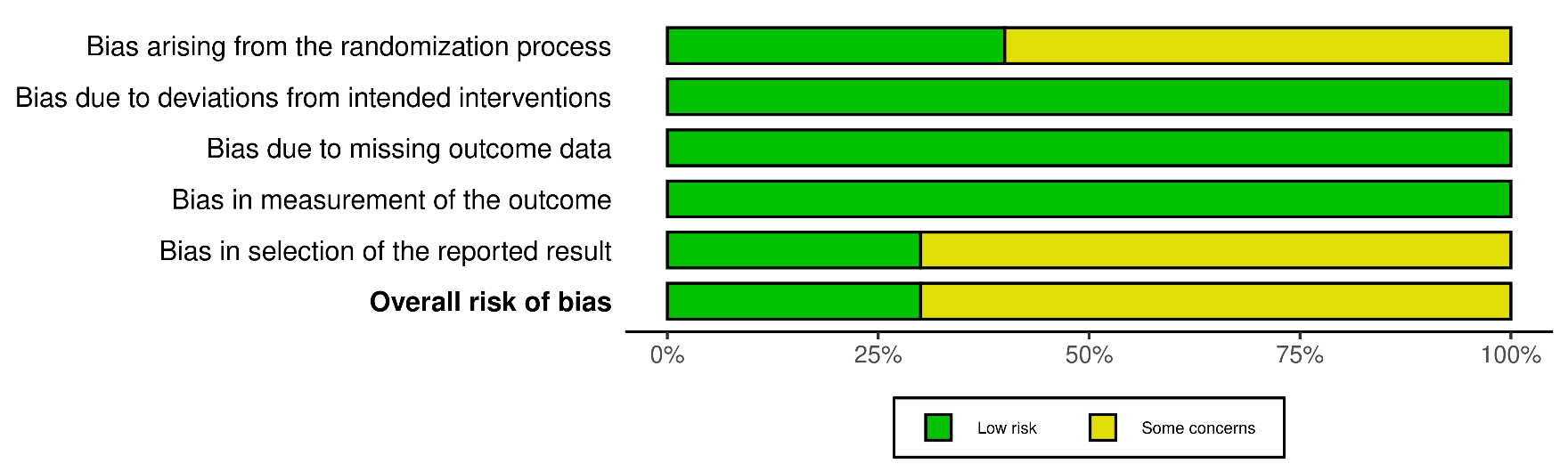 |

**Figure S6.** The risk of bias assessment at study and at domain level for the rebleeding rate

| 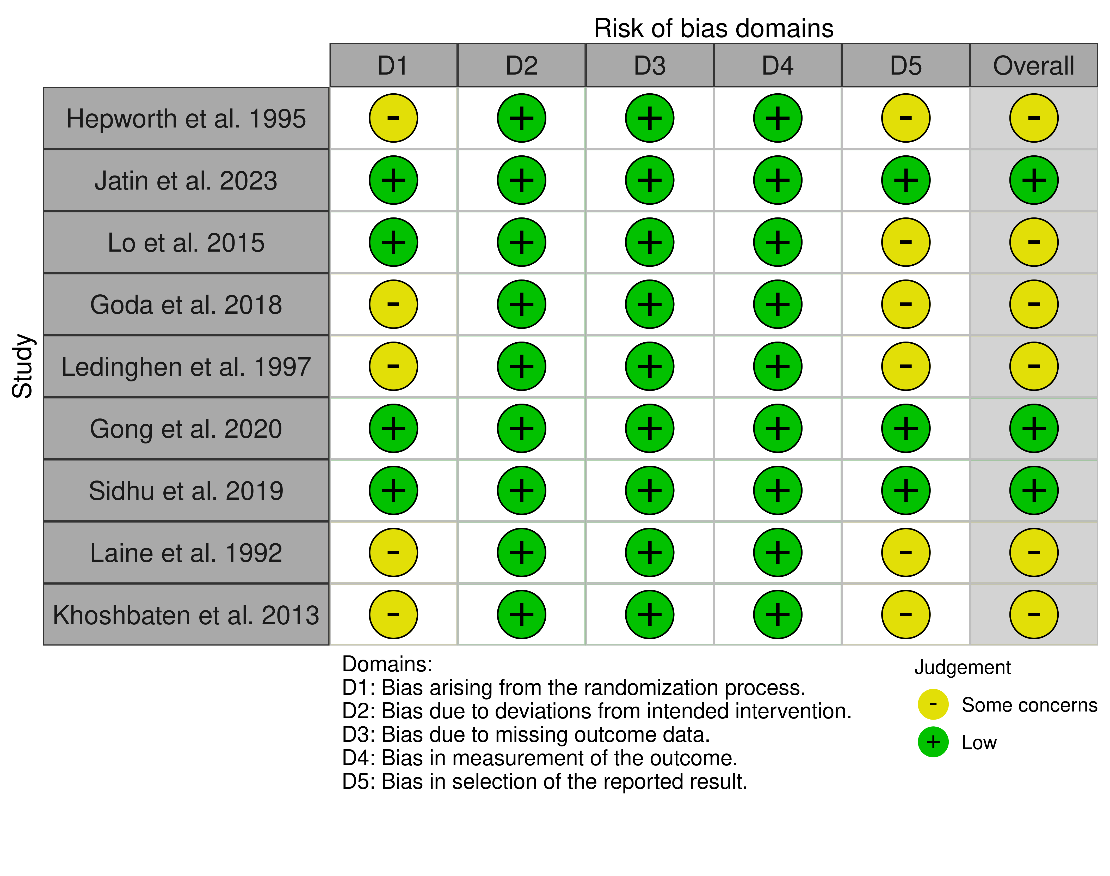 |
| --- |
| 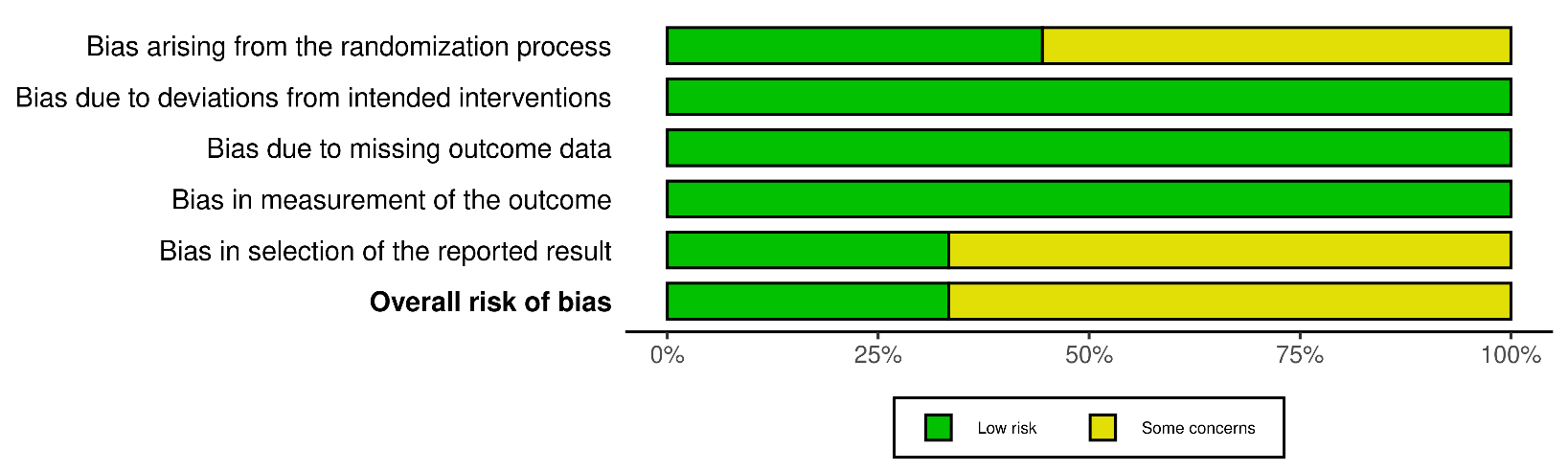 |

**Figure S7.** The risk of bias assessment at study and at domain level for the mortality rate

| 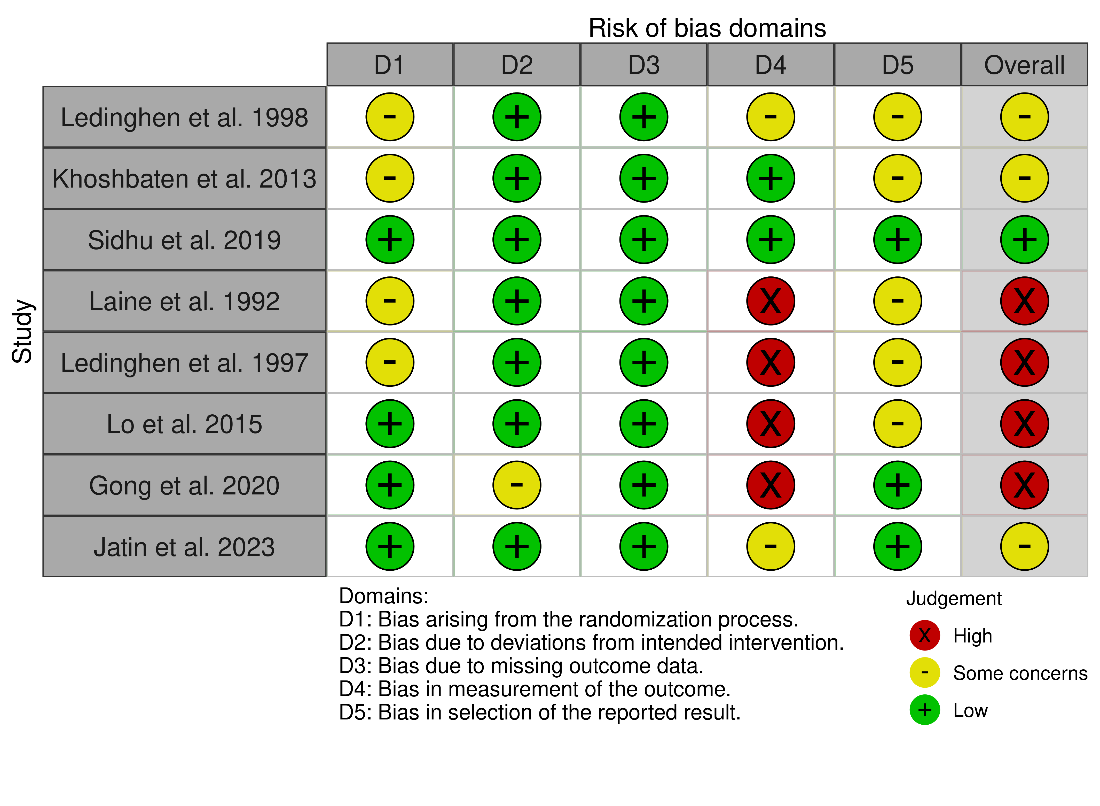 |
| --- |
| 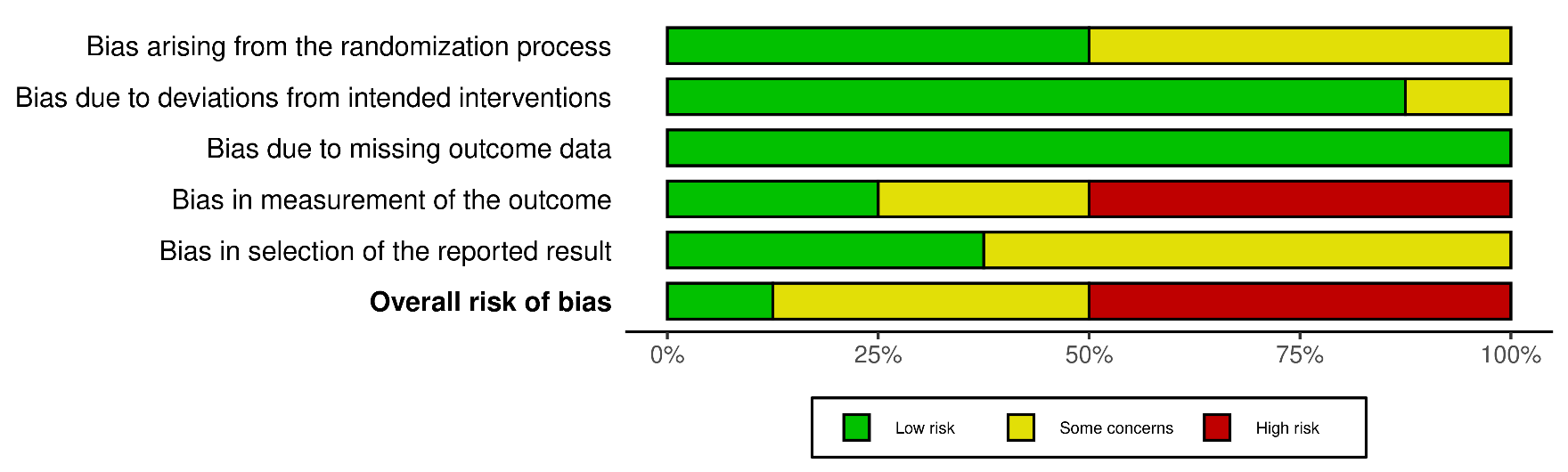 |

**Figure S8.** The risk of bias assessment at study and at domain level for the length of hospital stay

| 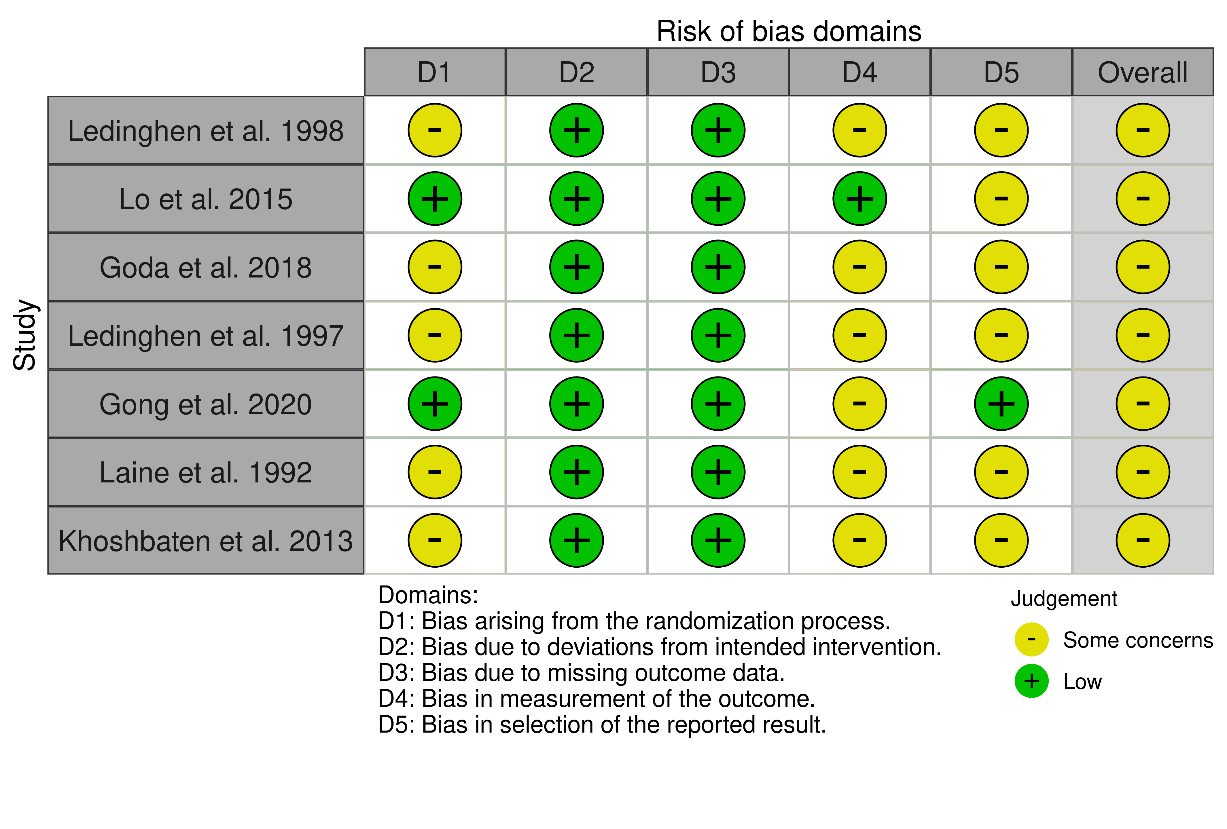 |
| --- |
| 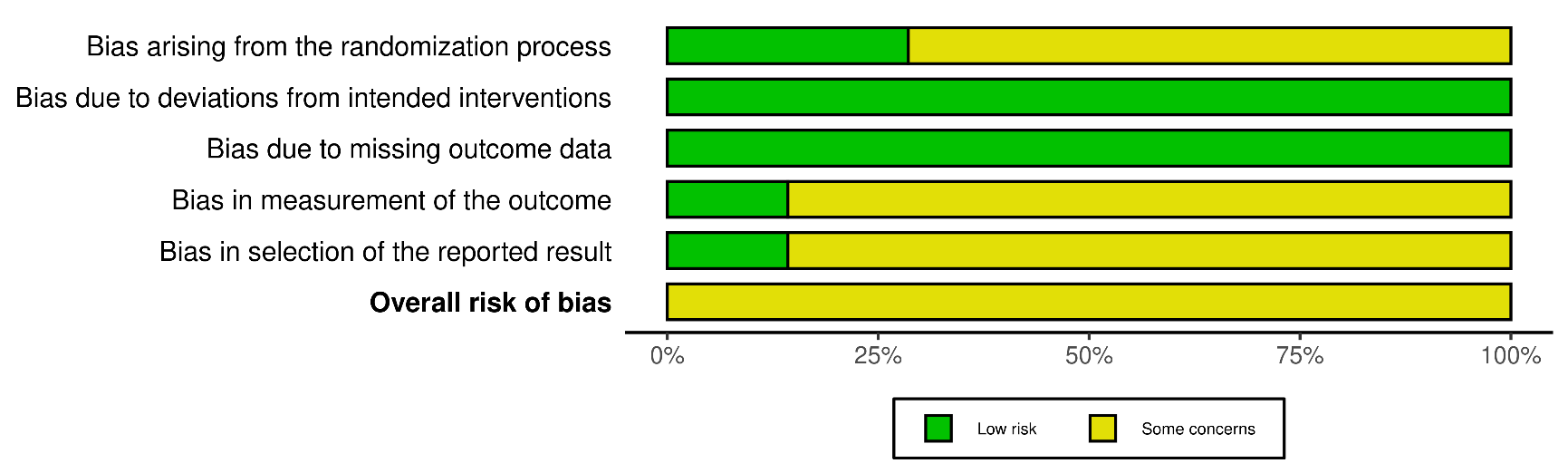 |

**Figure S9**. The risk of bias assessment at study and at domain level for blood transfusion requirement

| 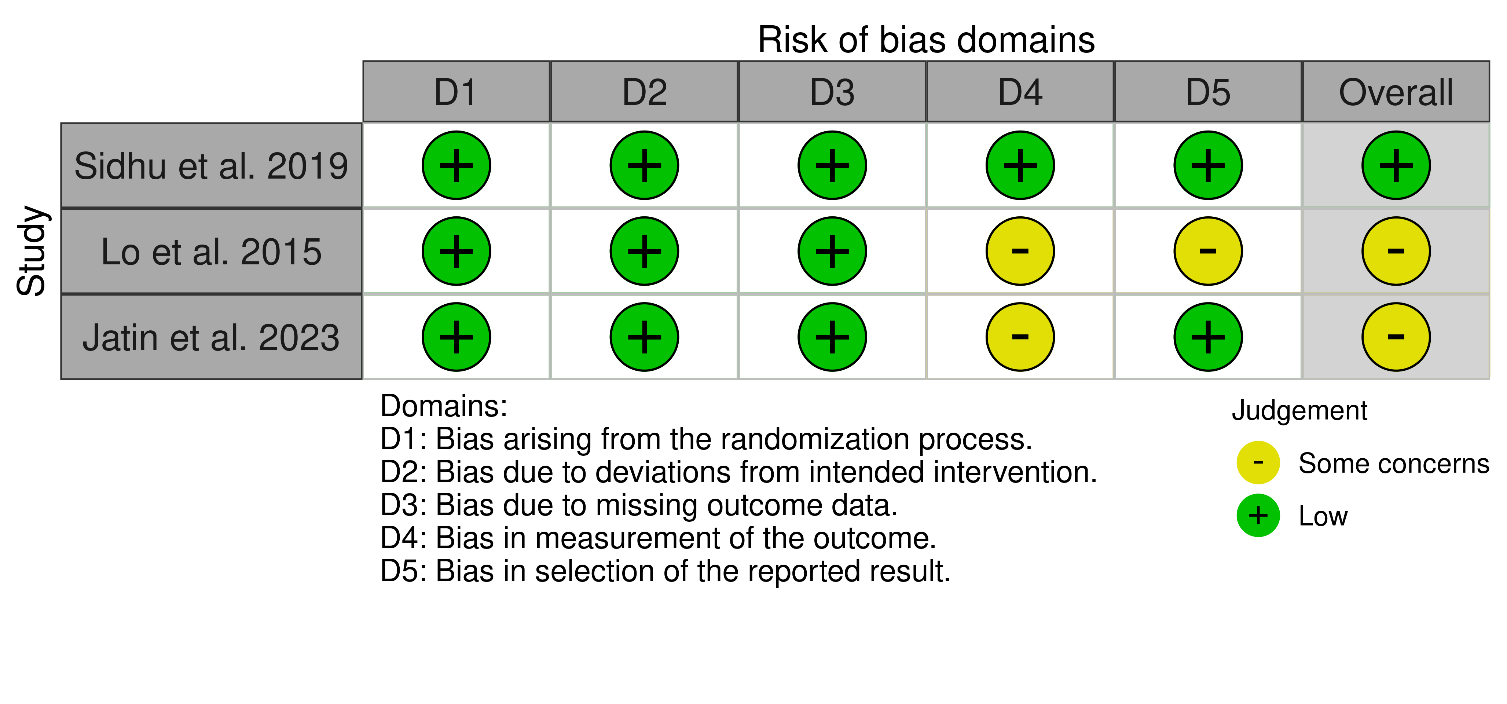 |
| --- |
| 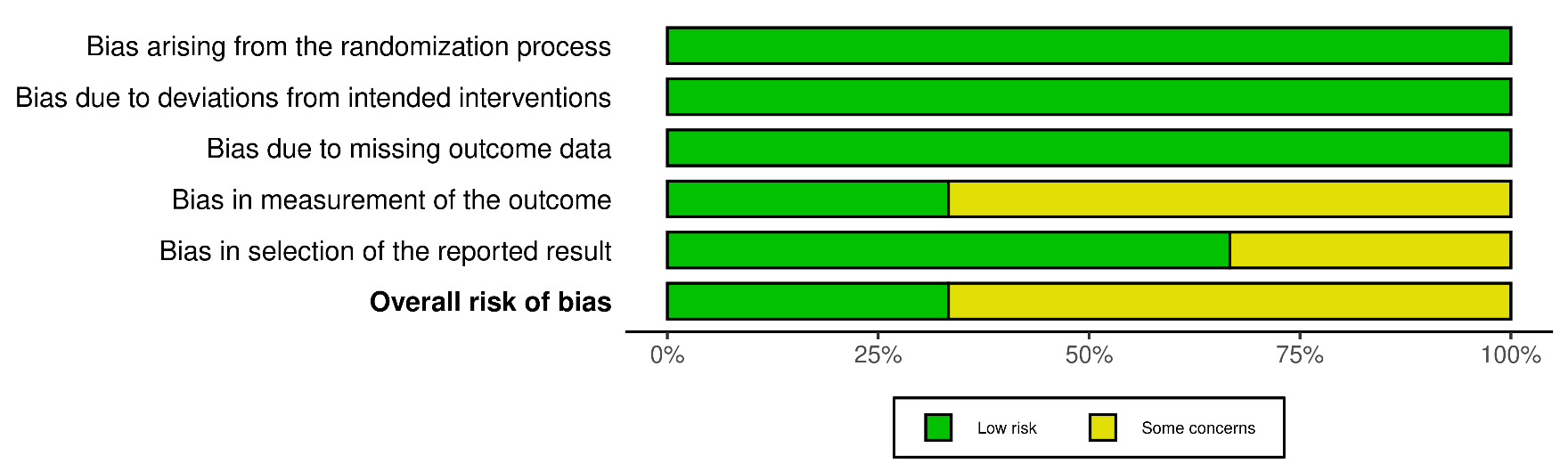 |

**Figure S10**. The risk of bias assessment at study and at domain level for bacterial infection

| 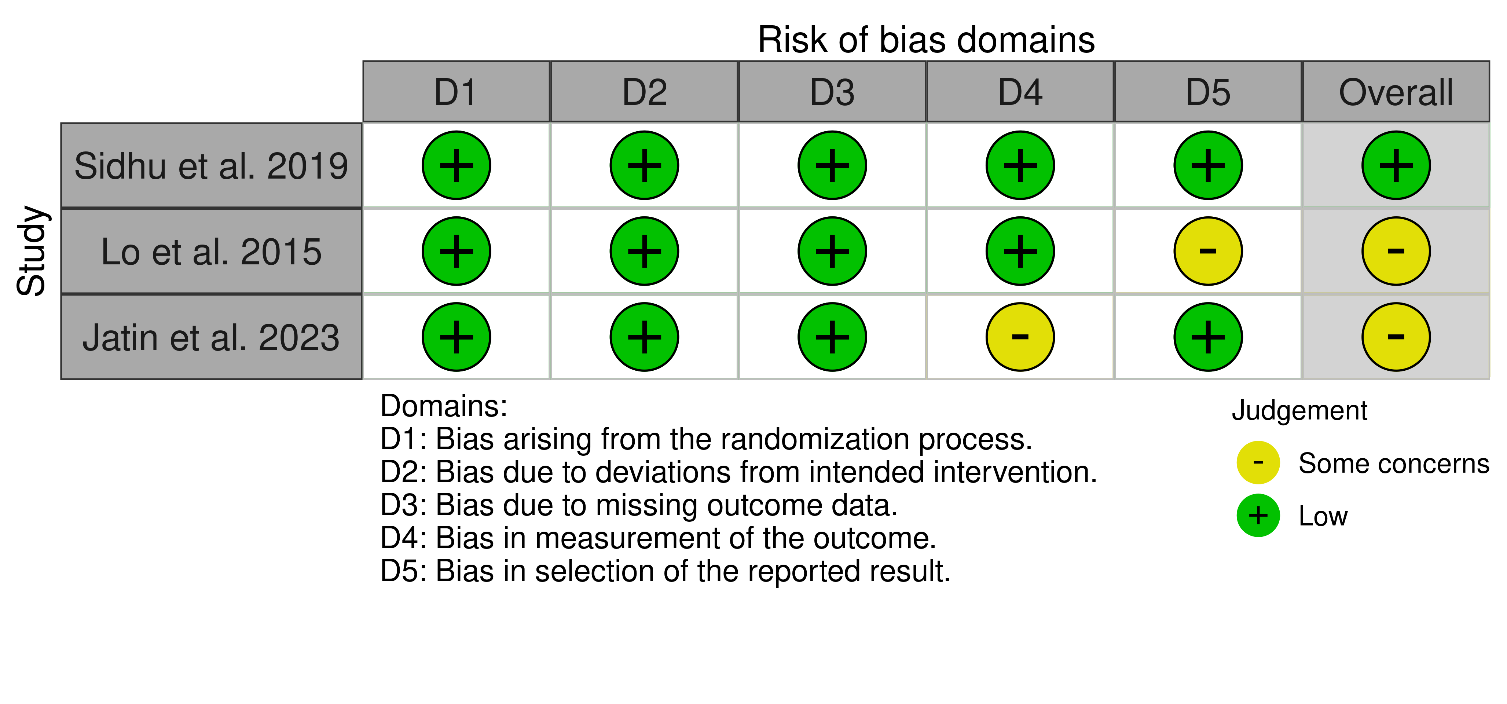 |
| --- |
| 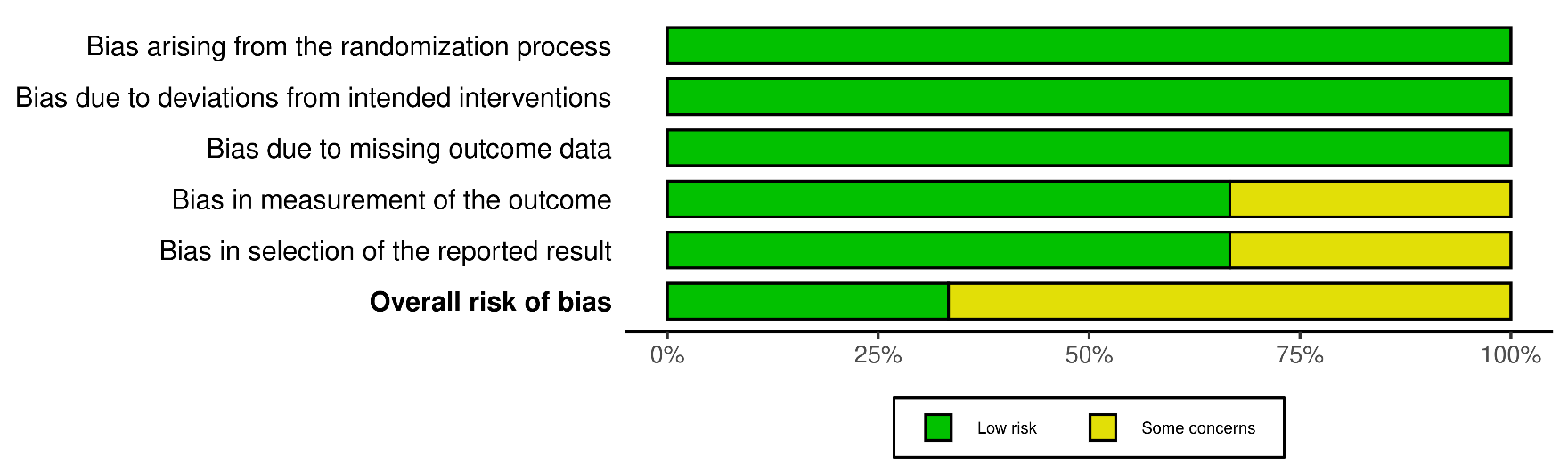 |

**Figure S11**. The risk of bias assessment at study and at domain level for the newly developed ascites

| 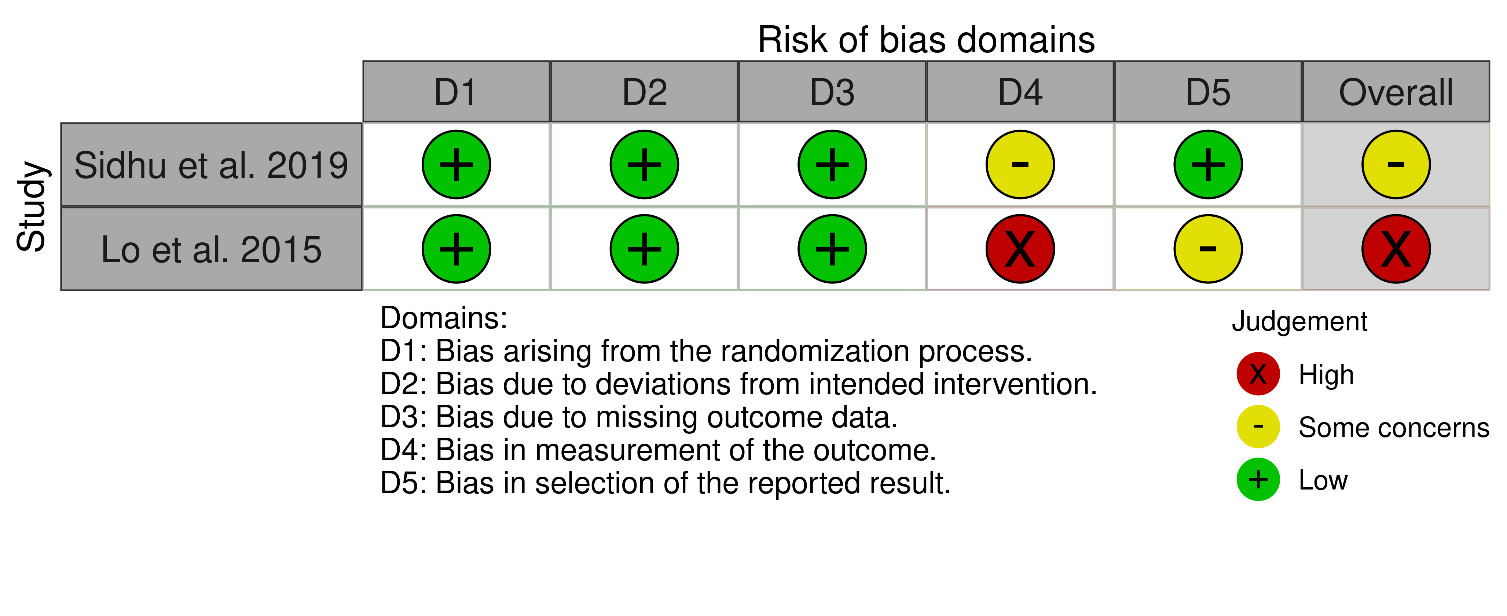 |
| --- |
| 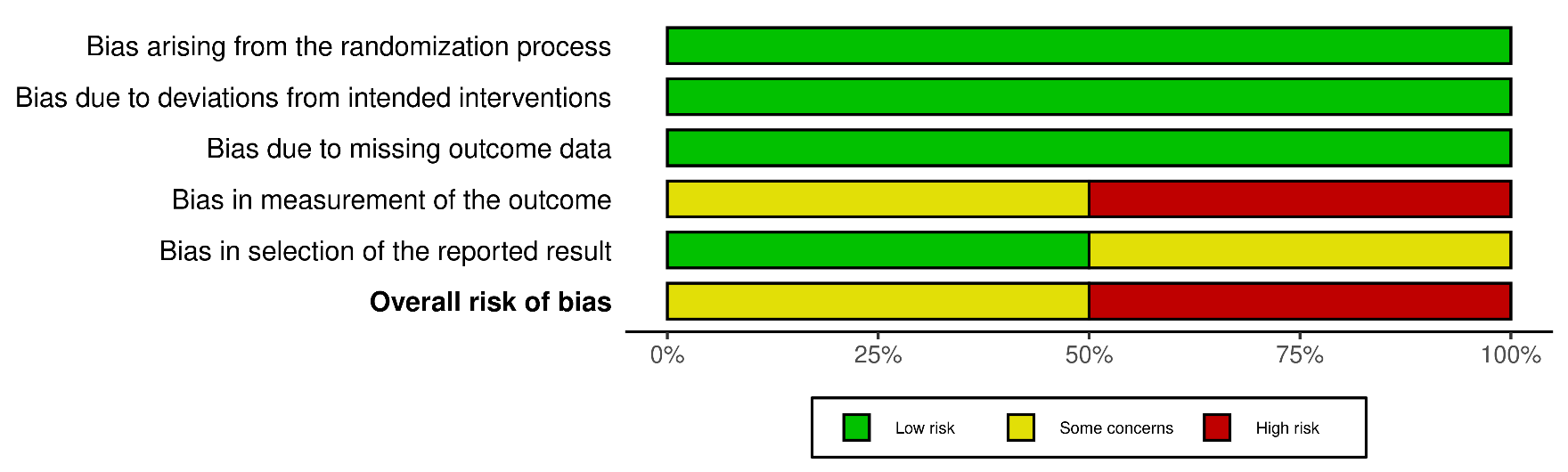 |

**Figure S12.** The risk of bias assessment at study and at domain level for the newly developed hepatic encephalopathy

**Table S4.** Summary of findings table of the quality of evidence of the rebleeding, mortality and length of hospital stay

| **Certainty assessment** | | | | | | | **№ of patients** | | **Effect** | | **Certainty** | **Importance** |
| --- | --- | --- | --- | --- | --- | --- | --- | --- | --- | --- | --- | --- |
| **№ of studies** | **Study design** | **Risk of bias** | **Inconsistency** | **Indirectness** | **Imprecision** | **Other considerations** | **Early Refeeding** | **Delayed Refeeding** | **Relative (95% CI)** | **Absolute (95% CI)** |  |  |
| **Early Rebleeding (less than 7 days)** | | | | | | | | | | | | |
| 8 | randomized trials | serious^a^ | not serious^b^ | serious^c^ | serious^d^ | none | 24/465 (5.2%) | 21/458 (4.6%) | **RR 1.04** (0.66 to 1.63) | **2 more per 1,000** (from 16 fewer to 29 more) | ⨁◯◯◯ Very low | CRITICAL |
| **Late Rebleeding (within 30-42 days)** | | | | | | | | | | | | |
| 8 | randomized trials | serious^a^ | not serious^b^ | serious^c^ | serious^d^ | none | 25/347 (7.2%) | 22/346 (6.4%) | **RR 1.16** (0.63 to 2.13) | **10 more per 1,000** (from 24 fewer to 72 more) | ⨁◯◯◯ Very low | CRITICAL |
| **Early Mortality (less than 7 days)** | | | | | | | | | | | | |
| 5 | randomized trials | serious^e^ | not serious^b^ | serious^c^ | serious^f^ | none | 3/274 (1.1%) | 2/269 (0.7%) | **RR 1.20** (0.85 to 1.71) | **1 more per 1,000** (from 1 fewer to 5 more) | ⨁◯◯◯ Very low | CRITICAL |
| **Late Mortality (within 30-42 days)** | | | | | | | | | | | | |
| 7 | randomized trials | serious^g^ | not serious^b^ | serious^c^ | serious^h^ | none | 17/335 (5.1%) | 29/332 (8.7%) | **RR 0.61** (0.36 to 1.06) | **34 fewer per 1,000** (from 56 fewer to 5 more) | ⨁◯◯◯ Very low | CRITICAL |
| **Length of Hospital Stay (days)** | | | | | | | | | | | | |
| 6 | randomized trials | serious^i^ | serious^j^ | serious^c^ | not serious | none | 289 | 281 | - | MD **1.22 days fewer** (2.43 fewer to 0.01 fewer) | ⨁◯◯◯ Very low | CRITICAL |

**CI:** confidence interval; **MD:** mean difference; **RR:** risk ratio

#### Explanations

a. There were some concerns about the risk of bias in most of the studies included for the analysis of this outcome. These mostly arose from the lack of details about the randomization process; however, there were no significant differences between study arms to suggest a problem with the randomization. Additionally, there was no pre-published protocol for most of the studies. Therefore, the authors decided to downgrade the level of evidence by one level.

b. The analysis of this outcome showed no unexplained heterogeneity.

c. Given the fact that there were widely varying definitions for the intervention and control groups across included studies, the authors decided to downgrade the level of evidence.

d. Considering that the 95% CI fails to exclude important benefit or important harm, the authors decided to rate down for imprecision.

e. There were some concerns about the risk of bias in most of the included studies. These arose from the lack of details about the randomization process. Additionally, there was no pre-published protocol for the studies. Therefore, a decision was made to downgrade the level of evidence for this outcome.

f. The number of participants analyzed (<500) and the total number of events (5 deaths) were very low. Therefore, the authors decided to downgrade the level of evidence.

g. There were some concerns regarding the risk of bias in most of the included studies. The potential for bias arose from the lack of details reported about the randomization process. In most studies there were no significant differences between the two study arms to suggest an issue with randomization. Additionally, there was no pre-published protocol for some of the included studies. Therefore, a decision was made to downgrade the level of evidence for this outcome by one level.

h. Given the fact tha the 95% CI includes the RR 1.00; and the number of analyzed participants is rather small, the authors decided to rate down one level for imprecision.

i. There were no clear clinical or biological criteria for patient discharge; additionally, there is no information regarding the blinding of the treating physicians to the assigned treatments in most of the studies. Therefore, the authors decided to downgrade the level of evidence.

j. The analysis of this outcome found significant heterogeneity. The leave-one-out sensitivity analysis found that the results of the article by Laine et al. (1992) may be the cause for heterogeneity. This article had a slightly different patient population as it only included patients with a non-bleeding Mallory-Weiss lesion or ulcer with a white base, which are now recognized as being at a low risk for rebleeding. Omission of this article resulted in much smaller heterogeneity.

**Table S5.** Summary of findings table of the quality of evidence of the blood transfusion requirement, bacterial infection, and new-onset ascites outcomes

| **Certainty assessment** | | | | | | | **№ of patients** | | **Effect** | | **Certainty** | **Importance** |
| --- | --- | --- | --- | --- | --- | --- | --- | --- | --- | --- | --- | --- |
| **№ of studies** | **Study design** | **Risk of bias** | **Inconsistency** | **Indirectness** | **Imprecision** | **Other considerations** | **Early Refeeding** | **Delayed Refeeding** | **Relative (95% CI)** | **Absolute (95% CI)** |  |  |
| **Transfusion Requirement** | | | | | | | | | | | | |
| 4 | randomised trials | serious^a^ | not serious^b^ | serious^c^ | not serious | none | 292 | 286 | - | MD **0 0.00**  (0.04 lower to 0.05 higher) | ⨁⨁◯◯ Low | CRITICAL |
| **Bacterial Infections** | | | | | | | | | | | | |
| 3 | randomised trials | serious^a^ | not serious^b^ | serious^c^ | serious^d^ | none | 8/128 (6.3%) | 16/123 (13.0%) | **RR 0.48** (0.08 to 3.05) | **68 fewer per 1,000** (from 120 fewer to 267 more) | ⨁◯◯◯ Very low | CRITICAL |
| **Newly Developed Ascites** | | | | | | | | | | | | |
| 3 | randomised trials | serious^a^ | not serious^b^ | serious^c^ | serious^d^ | none | 12/128 (9.4%) | 18/123 (14.6%) | **RR 0.64** (0.34 to 1.20) | **53 fewer per 1,000** (from 97 fewer to 29 more) | ⨁◯◯◯ Very low | CRITICAL |

**CI:** confidence interval; **MD:** mean difference; **RR:** risk ratio

#### Explanations

a. In most or all the studies included in the assessment of this outcome there were some concerns regarding the risk of bias, therefore, the authors decided to downgrade the level of evidence.

b. There was no unexplained heterogeneity in the results regarding this outcome.

c. There were varying definitions of the two interventions (early versus delayed nutrition). Therefore, the authors decided to downgrade the level of evidence.

d. The number of studied participants and event numbers are relatively small, and the CI includes a widely varying interval. For these reasons, the authors decided to rate down by one level due to significant imprecision.


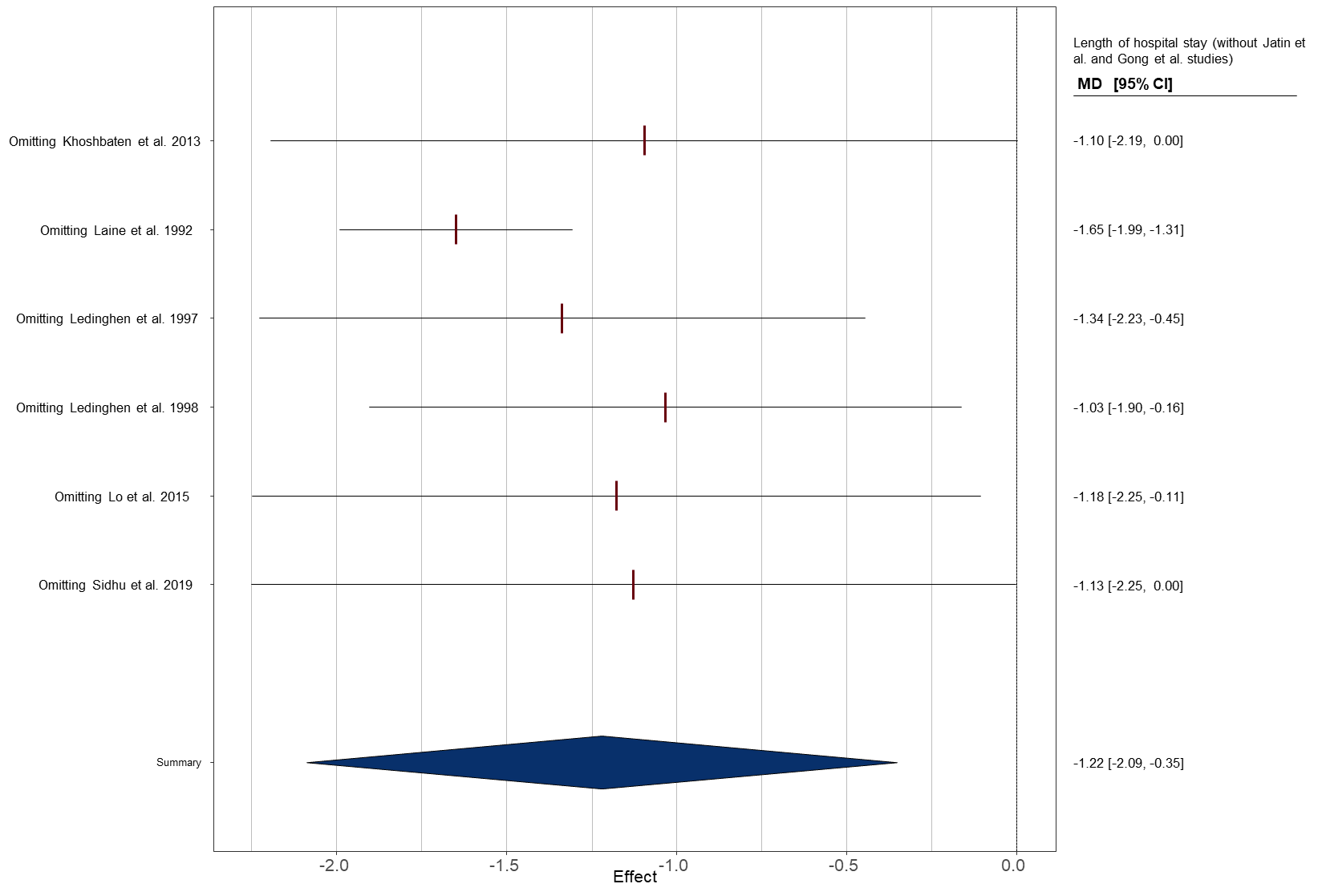


**Figure S13.** Leave-one-out sensitivity analysis in the length of hospital stay days based on the effect size. MD, mean difference; CI, confidence interval.

**
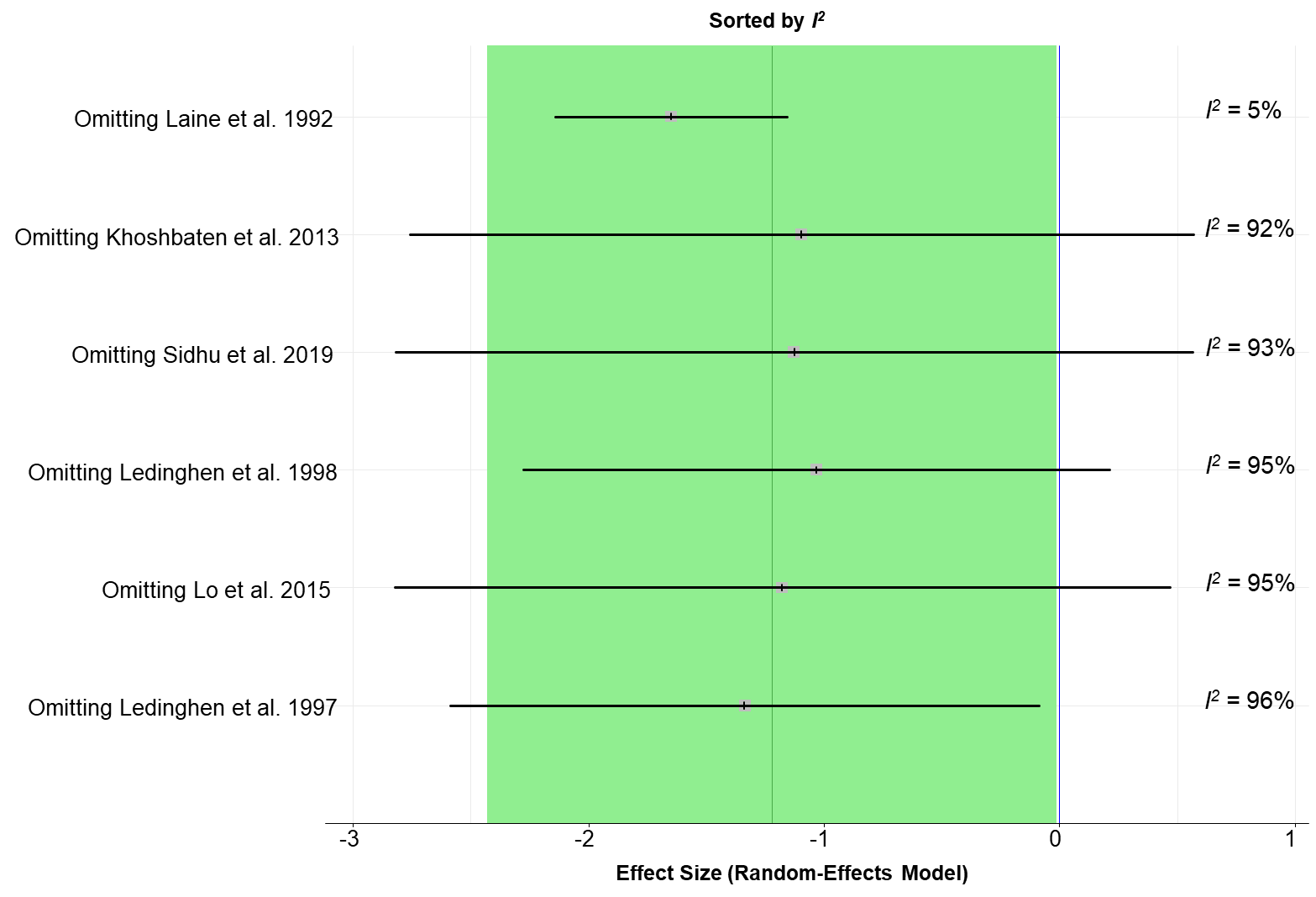
**

**Figure S14.** Leave-one-out sensitivity analysis in the length of hospital stay days sorted by the heterogeneity level.

| **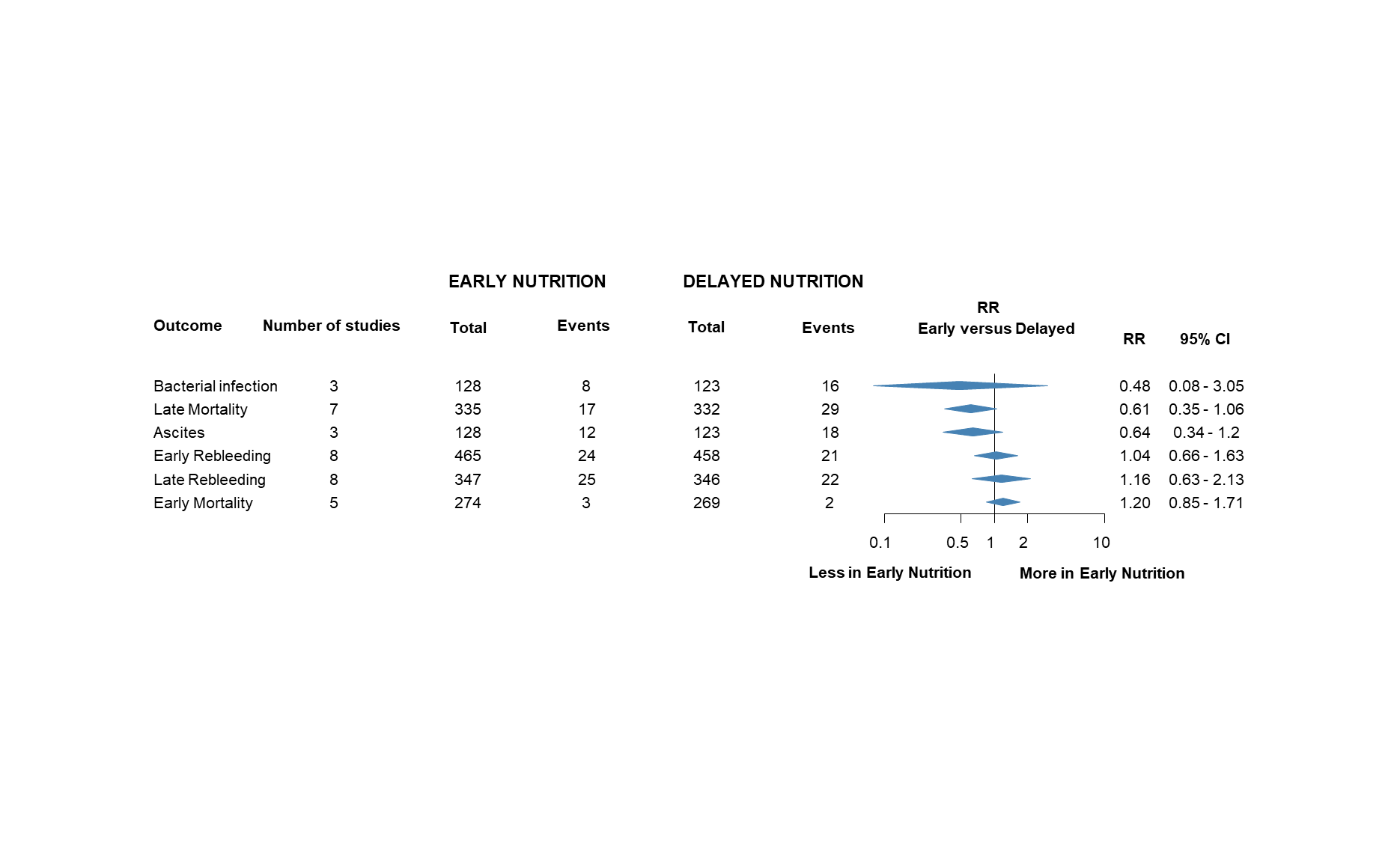A** |
| --- |
| **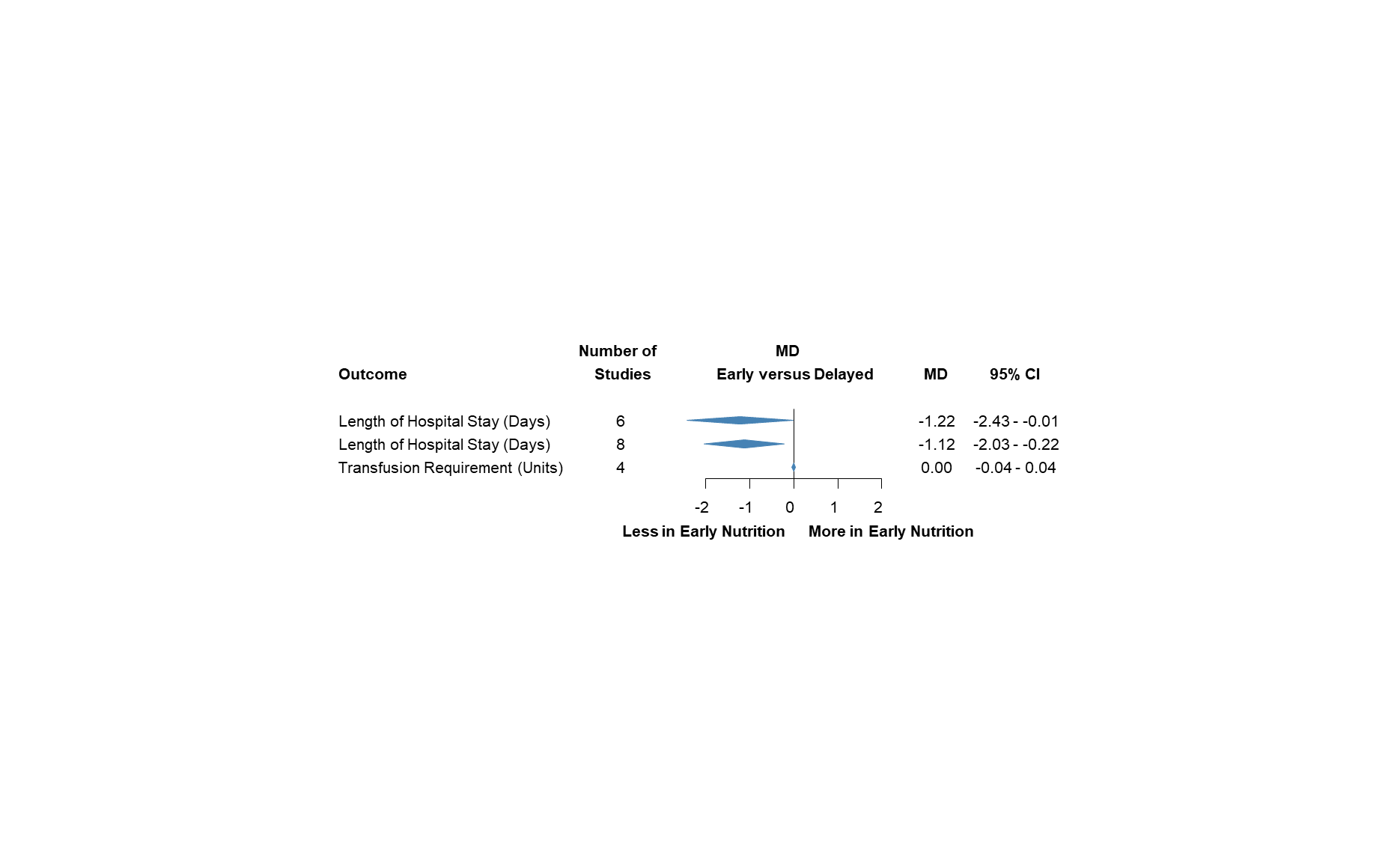B** |

**Figure S15.** Summary forest plots demonstrating the effect of early versus delayed nutrition in all the investigated outcomes. **A**. Binary outcomes. RR, risk ratio; CI, confidence interval. **B**. Continuous outcomes. MD, mean difference; CI, confidence interval**.**

**Table S6.** Detailed definitions of the timing and dietary nutrition in the included studies

| **Study (year)** | **Definition of Early Nutrition** | **Definition of Delayed Nutrition** |
| --- | --- | --- |
| Sidhu et al. 2019 ^6^ | Start liquid diet (plain water or fruit juice) after 1 h following EVL. After 4 h, a regular diet was resumed. | To fast for 4 h after endoscopic treatment. Thereafter, a liquid diet was given to patients for 24 h, after which a soft diet was provided for the next 48 h. After 72 h post-EVL, a regular diet was resumed. |
| Goda et al. 2018 ^8^ | Start oral feeding just 4 h after undergoing emergency endoscopic intervention. Regarding the type of diet commenced, patients in this group were allowed only a liquid diet such as milk, fruit juice, or rice in the liquid formula in the first 48 h. Solid foods were not permitted. In the following 3 days, they were allowed to start a soft diet, and then starting from the sixth day of endoscopy, they were provided a regular diet. | Completely withhold feeding for 2 days after emergency endoscopic management. Regarding nutritional supply during that period, they provided them with 1500 ml/day of parenteral fluids (0.9% saline and dextrose water) with adequate electrolytes, and this continued for 48h. Following 2 days of abstaining from eating, patients were commenced on a liquid diet for 1 day, and afterward, patients then were provided with a soft diet for the following 2 days, and eventually, they resumed a regular diet on the sixth day following emergency upper GI endoscopy. |
| Lo et al. 2015 ^1^ | They were asked to fast for only 4 hours following endoscopic treatment. Subsequently, a liquid diet (fruit juice, soybean juice, milk, rice in liquid form) was instituted for 3 days. Additionally, <500 cc intravenous fluid with proper electrolyte supplement per day was administered. Thereafter, a soft diet was provided for 3 days, after which a regular diet was resumed since the seventh day after endoscopic treatment. | They were asked to absolutely fast for 48 hours after endoscopic treatment, and 1500 cc/day intravenous fluids (normal saline or glucose water) with proper electrolytes were administered for 2 days. After 2 days of fasting, a liquid diet was given for 1 day, and subsequently, a soft diet was given for 3 days, and then a regular diet was instituted on the seventh day after endoscopic treatment. |
| Ledinghen et al. 1997 ^3^ | Polymeric diet within 24 h (Dripac Sondalis, Sopharga, France) was infused by bolus administration and provided 1665 kcal/day and 71 g of protein. A constant-infusion pump delivered each Dripac in 3 h, via an 10 French nasogastric feeding tube. | On day 4, all patients received a standard low-sodium milk diet (800 kcal), on day 5 a mixed, warm, low- sodium diet (1400 kcal), and on day 6 a standard low- sodium hospital diet (1800 kcal). |
| Jatin et al. 2023 ^9^ | A liquid diet was started one hour later which consisted of commercial polymeric formula feed for the first 6 hours followed by homemade milk-based formula feed. A solid diet was resumed 48 hours later in both groups. | Only sips of water and lemon water orally for 48 hours after endotherapy. Solid diet was resumed after 48 hours. |
| Gong et al. 2020 ^7^ | Feeding was initiated 24h after hemostasis. Initially, sips of water were permitted for the first 6 hours, followed by a liquid diet that did not require chewing (e.g., soup) for 24 hours. A soft diet (e.g., rice gruel) was then administered for 24 hours, and a regular diet was administered thereafter. | Feeding was initiated 48h after hemostasis. Initially, sips of water were permitted for the first 6 hours, followed by a liquid diet that did not require chewing (e.g., soup) for 24 hours. A soft diet (e.g., rice gruel) was then administered for 24 hours, and a regular diet was administered thereafter. |
| Khoshbaten et al. 2013 ^2^ | Patients were removed from nil-per-os (NPO) between 6 and 12 h after endoscopic treatment and fed with a liquid diet consisting of soup (the food available at the hospital). | Patients were prevented from oral feeding for 72 hours after endoscopy and had dextrose saline intravenous fluids. On the third day, endoscopy was done again and the existence of new wounds and new pathological findings were recorded on the checklist. If patients had any signs of bleeding, they remained at NPO. |
| Laine et al. 1992 ^4^ | Immediate refeeding group (regular diet immediately after endoscopy). | Nothing by mouth for 36h, followed by a clear liquid diet for 12h and then a regular diet 48h after entry. |
| Ledinghen et al. 1998 ^5^ | Received milk on day 1, mixed warm feeding on day 2 and normal diet from day 3. | Nil by mouth until day 3, then received milk on day 4, mixed warm feeding on day 5, and normal diet from day 6. |
| Hepworth et al. 1995 ^10^ | Receive normal diet and 50mls milk 2 hourly. | Nil by mouth for 24 hours. |

**REFERENCES**

1 Lo, G. H., Lin, C. W. & Hsu, Y. C. A controlled trial of early versus delayed feeding following ligation in the control of acute esophageal variceal bleeding. *Journal of the Chinese Medical Association : JCMA* **78**, 642‐647 (2015). <https://doi.org:10.1016/j.jcma.2015.07.004>

2 Khoshbaten, M., Ghaffarifar, S., Jabbar Imani, A. & Shahnazi, T. Effects of early oral feeding on relapse and symptoms of upper gastrointestinal bleeding in peptic ulcer disease. *Digestive endoscopy* **25**, 125‐129 (2013). <https://doi.org:10.1111/j.1443-1661.2012.01347.x>

3 de Lédinghen, V. *et al.* Early feeding or enteral nutrition in patients with cirrhosis after bleeding from esophageal varices? A randomized controlled study. *Digestive diseases and sciences* **42**, 536‐541 (1997). <https://doi.org:10.1023/a:1018838808396>

4 Laine, L. *et al.* Prospective evaluation of immediate versus delayed refeeding and prognostic value of endoscopy in patients with upper gastrointestinal hemorrhage. *Gastroenterology* **102**, 314‐316 (1992). <https://doi.org:10.1016/0016-5085(92)91816-m>

5 de Lédinghen, V. *et al.* When should patients with bleeding peptic ulcer resume oral intake? A randomized controlled study. *Gastroenterologie clinique et biologique* **22**, 282‐285 (1998).

6 Sidhu, S. S. *et al.* Early feeding after esophageal variceal band ligation in cirrhotics is safe: randomized controlled trial. *Digestive endoscopy* (2019). <https://doi.org:10.1111/den.13423>

7 Gong, E. J. *et al.* Optimal Timing of Feeding After Endoscopic Hemostasis in Patients With Peptic Ulcer Bleeding: a Randomized, Noninferiority Trial (CRIS KCT0001019). *American journal of gastroenterology* **115**, 548‐554 (2020). <https://doi.org:10.14309/ajg.0000000000000584>

8 Goda, T., Mokhtar, A.-R., Anwar, R., Hazem, H. & Eleraki, A. Effect of early versus delayed feeding following emergency endoscopic therapy for acute esophageal variceal bleeding on short-term outcomes. *The Egyptian Journal of Internal Medicine* **30**, 110-114 (2018). <https://doi.org:10.4103/ejim.ejim_22_18>

9 Jatin, Y. *et al.* An Open-label Randomized Controlled Trial of Early Initiation of Nasogastric Feeding After Endotherapy in Variceal Bleeding: A Proof-of-concept Study. *Journal of Clinical and Experimental Hepatology* (2023). <https://doi.org:https://doi.org/10.1016/j.jceh.2023.07.413>

10 Hepworth, C. C. *et al.* RANDOMIZED CONTROLLED TRIAL OF EARLY FEEDING IN PATIENTS WITH BLEEDING PEPTIC-ULCER AND A VISIBLE VESSEL. *Gastroenterology* **108**, A113-A113 (1995).
